# Correction: *Magnitude and modifiers of the weekend effect in hospital admissions: a systematic review and meta-analysis*

**DOI:** 10.1136/bmjopen-2018-025764corr1

**Published:** 2019-06-27

Chen Y, Armoiry X, Higenbottam C, *et al*. Magnitude and modifiers of the weekend effect in hospital admissions: a systematic review and meta-analysis. *BMJ Open* 2019;9:e025764. doi: 10.1136/bmjopen-2018-025764.

This article was previously published with an error.

The author name ‘Rajna Basra’ should be ‘Ranjna Basra’.

