# Supplementary material for: Magnitude and modifiers of the weekend effect in hospital admissions: a systematic review and meta-analysis
Source: BMJ Open. 2019 Jun 4;9(6):e025764. doi: 10.1136/bmjopen-2018-025764 (PMC6561443; doi:10.1136/bmjopen-2018-025764)
Supplement: Supplementary file 1 [file bmjopen-2018-025764supp001.pdf]

The magnitude of the weekend effect in hospital admissions: a systematic review and meta-analysis

Yen-Fu Chen, Xavier Armoiry, Caroline Higenbottam, Nick Cowley, Ranjna Basra, Samuel Watson, Carolyn Tarrant, Amunpreet Boyal, Elizabeth Sutton, Chia-Wei Wu, Cassie Aldridge, Amy Gosling, Richard Lilford, Julian Bion, on behalf of the HiSLAC collaboration.

Supplementary file: online appendices

Please note that references cited in this supplementary file are listed at the end of this document. Therefore the reference numbers cited in-text correspond to reference numbers of the reference list within this supplementary file, and they are different from the reference numbers quoted in the main paper.

Contents

Appendix 1. Data extraction and coding manual for the systematic review 3
Appendix 2. Risk of bias assessment 15
Appendix 3. Rationale and technical details of Bayesian analyses 16
3.1 Rationale for undertaking Bayesian meta-analysis and meta-regression 16
3.2 Technical details of the Bayesian meta-analysis 16
3.3 Technical details of the Bayesian meta-regression 17
Appendix 4. Examination of potential overlap in the coverage of admissions between different studies 19
4.1 Potential overlap in data between studies of hospital mortality in England 20
4.2 Potential overlap in data between studies of hospital mortality in the USA 24
Appendix 5. PRISMA flow diagram 28
Appendix 6. Characteristics of included studies (sorted by type of admissions) 29
Appendix 7. Technical information for Bayesian meta-analysis 36
7.1 Trace-plots and pairs plots for primary Bayesian meta-analysis 36
7.2 Statistical outputs for primary Bayesian meta-analysis and sensitivity analysis 38
7.3 Statistical outputs for Bayesian subgroup analyses 39
Appendix 8. Sensitivity analyses 41
8.1 Sensitivity analyses for the primary meta-analysis 41
Appendix 9. Subgroup analyses (mortality) 44
9.1 Subgroup analyses by types of admissions 44
9.1.1 All admissions 44

9.1.2 Emergency admissions 45
9.1.3 Elective admissions 46
9.1.4 Maternity admissions 47
9.1.5 Within study comparisons: emergency vs elective admissions 47
9.1.6 Emergency admissions through Accident & Emergency (A&E) department 48
9.2 Mortality – subgroups by time period 49
9.3 Mortality – subgroups by country 50
9.4 Mortality – subgroups by disease conditions 51
9.5 Mortality – correlation of hospital weekend staffing level and the weekend effect 54
Appendix 10. Impact of variations in methodological approaches 56
10.1 Impact of statistical adjustment for acute physiology 56
10.2 Different definitions of weekends 57
10.3 Different measures for mortality 57
10.4 Different effect measures 59
10.5 Multiple analyses of the same or overlapping data set(s) 60
Appendix 11. Evidence on the weekend effect related to adverse events 62
11.1 Composite measures of adverse events 62
11.2 Needs for further hospital care following initial admission 64
11.3 Patient safety indicators and surgical adverse events 67
11.4 Perinatal and neonatal adverse events 71
11.5 Maternal adverse events 73
Appendix 12. Evidence on the weekend effect related to length of stay (LOS) 75
Appendix 13. Evidence on the weekend effect related to patient satisfaction 80
Appendix 14. GRADE assessment for overall quality of evidence 82
References for appendices 86

Appendix 1. Data extraction and coding manual for the systematic review

Below is a list of data items that we want to extract from included studies into the Excel spreadsheet provided. Please follow the instructions/examples as closely as possible when you go through each item.

Some items require free text while others require some sort of classification / coding. For the latter the *codes* are listed in the table below. If none of the *codes* seems to be appropriate, you can always code it as '*Other'* (when none of the codes is suitable) or 'Unclear' (when you are not sure which codes to choose) and then put further details using the 'Comment' function (you can do this by firstly select the relevant cell, then right click and choose the 'Insert comment' option).

When the desired information was not described/reported in the paper, please code as 'NR' (not reported). Sometimes an item is not relevant for a particular study, in which can you can enter 'NA' (not applicable).

**Item**	**Free text to enter / codes**	**Explanation**
**Study characteristics and methods**		
**Author year** (Free text)	First author and year of publication<br>e.g. Albright 2009	The first author's last name and year of publication of the paper.
**ID**<br>(Number)	The record number for the EndNote database	This is provided in the file name of the paper.
**Extracted**<br>(Reviewer initials DD/MM/YY)	Identity of the reviewer carrying out data extraction and the date when it is carried out	Please enter your initials and date on which data extraction for this study was completed, e.g. XA 19/04/16
**Checked** (Reviewer initials DD/MM/YY)	Identity of the reviewer carrying out data checking and the date when it is carried out	Please leave this blank (to be completed during data checking)
**Further comments**<br>(Free text)	Free space	This is a free space for you to add any comments and observations not captured in the extracted data or raise any questions to be discussed
**Country**<br>(Free text)	Name of country or region<br>e.g. USA; six Middle Eastern countries	The name of the country/countries where the study was conducted. Further information on region/location can be entered as comments

**Study period**<br>(free text)	Year(s) for which data were collected<br>e.g. 2001; 1994 – 2002	Please record year(s) and months (where reported)
**Data source**<br>(free text)	List the source of the data, e.g. HES, HCUP NIS; or code as<br>• *ad hoc*	Please record the name of the database/registry/audit - either abbreviation (if available – please record full name in the cell comment) or full name; or code as 'ad hoc' which indicates that the data was collected specifically for a study without a study name
**Type of data source**<br>(code)	• *Administrative*<br>• *Clinical*	Code as *'Administrative'* if the data came from a routine database such as HES in England and NIS in the US; code as *'clinical'* if the data came from ad hoc registry or audit in which clinical information was also collected.
**Accuracy of data source**<br>(free text)	List information concerning the accuracy and completeness of the data source	This is usually in the form of previous studies (e.g. comparison of coding accuracy). If no information was provided, state "NR".
**Inclusion/exclusion criteria**<br>(free text)	Enter (copy & paste) the criteria for selecting patients / admissions into the study and the rationale behind the criteria (if provided)	Record "NR" where applicable.
**Cross-sectional or longitudinal (type of data)**<br>(*code*)	• *Cross-sectional*<br>• *Longitudinal*<br>• *Both*	Code as '*Cross-sectional'* if the data were analysed as one period (irrespective of whether it spanned over several years); code as '*Longitudinal'* if data were collected and analysed for more than one year (e.g. repeated cross-sectional data by years) and allowed the observation of changes over years. Can code 'Both' if both an overall estimate and a break down result by years are reported.
**Nature of admission:**<br>➢ **Emergency**<br>➢ **Elective**<br>➢ **Maternity**<br>(*code*)	• *Yes*<br>• *No*<br>• *Unclear (please explain)*	Code as '*Yes', 'No' or 'Unclear' for each type of the admissions.*<br>If the study include all hospital admissions without specific inclusion/exclusion criteria, code '*Yes'* to all three types of admission.
**Procedures involved:**<br>➢ **Medical**<br>➢ **Surgical**<br>➢ **Childbirth**<br>(*code*)	• *Yes*<br>• *No*<br>• *Unclear (please explain)*	Code as '*Yes', 'No' or 'Unclear' for each main type of procedures involved in the admissions.*<br>If the study include all hospital admissions without specific inclusion/exclusion criteria, code '*Yes'* to all three types of admission.

**Type of patients:**<br>➢ **Adult**<br>➢ **Paediatric**<br>➢ **Maternity**<br>(*code*)	• *Yes*<br>• *No*<br>• *Unclear (please explain)*	Code as '*Yes', 'No' or 'Unclear' for each main type of procedures involved in the admissions.* If the study include all hospital admissions without specific inclusion/exclusion criteria, code '*Yes'* to all three types of admission.
**Comparison**<br>➢ ***Weekend vs weekday***<br>➢ ***Out-of-hours vs regular hours***<br>(*code*)	• *Yes*<br>• *No*<br>• *Unclear (please explain)*	Choose the most appropriate code or code '*Other'* and record further details in the cell comment.
**Definition of weekend /weekday (and/or out-of-hours)**<br>(free text)	Please record (copy & paste) the definition(s)	e.g. weekend was defined as from xx hour on Friday to xx hour on Monday; whether other public holidays were included.
**Reference day/time and rationale**<br>(free text)	Please record the reference day (time period) used to estimate the weekend effect (and the rationale if stated)	If more than one reference day or time period (against which weekend admissions were compared) was used, please record all (and where reported, which was used in the primary analysis, the rationale and whether this was pre-specified).
**Sensitivity analyses by using different reference day/time**<br>(code)	• *Yes*<br>• *No*	Code '*Yes'* if the study had estimated weekend effects using more than one reference day/time<br>Otherwise code *'No'*
**Subgroup analyses by condition(s)**<br>(*code*)	• *Yes*<br>• *No*	Code '*Yes'* if the study reported weekend effects for specific conditions/diagnoses in addition to an estimate for all admissions<br>Otherwise code *'No'*
**Additional analyses**<br>(free text)	List any other comparisons or analyses that were carried out	For example additional comparisons between night time vs day time; analyses based on different definitions of weekends or outcomes (e.g. 7-day mortality vs 30-day mortality);analyses of mortality risk by number of days since admission; etc
**Final sample size**<br>(number)	List the total sample size in terms of number of admissions	Final sample size is defined here as the number of admissions included in the analysis. If the unit was the number of patients, highlight this in the cell comment.
**Initial sample size**<br>(number)	List the initial sample size before any exclusions were made; or code<br>• No exclusion<br>• NR	Initial sample size is defined as the number of admissions included in the initial sample before any exclusion (e.g. due to incomplete data) was made.
**Number of hospitals**<br>(number)	List the number of hospitals from which the admissions were sampled	Record the number of hospitals and put additional information (such as the number of NHS Trusts) in *Comment*

**Mortality**<br>(*code*)	• *Yes*<br>• *No*	Code '*Yes*' if the study examined weekend effect on mortality and '*No*' if it did not.
**Mortality definition**<br>(free text)	Record how mortality was defined/ measured in the study, e.g. in-hospital and 30-day	Please record all measures if there is more than one, e.g. in-hospital mortality and 90-day mortality.
**Adverse events (AEs)**<br>(code)	• *Yes*<br>• *No*	Adverse events (AEs) are defined here as any undesirable events (other than death) that may be caused by medical management rather than the underlying condition of the patient, e.g. surgical complications. This definition does not imply preventability.<br><br>Interventions and procedures that are carried out mainly to deal with AEs rather than as part of the routine management of a condition are sometime used as indicators for the occurrence of AEs, such as some of the items included in the Patient Safety Indicators. These will also be considered as AEs for this review.<br><br>Code '*Yes*' if the study examined weekend effect on AEs and '*No*' if it did not.
**AE definition**<br>(free text)	Record what AE(s) were examined and their definition(s)	Include methods for identifying AEs where relevant (e.g. using ICD codes or review of case notes etc.)
**Length of stay (LoS)**<br>(*code*)	• *Yes*<br>• *No*	Code '*Yes*' if the study examined weekend effect on the length of stay in the hospital and '*No*' if it did not.
**LoS definition**<br>(free text)	Record how LoS was estimated	Record 'NR' where appropriate.
**Patient satisfaction**<br>(*code*)	• *Yes*<br>• *No*	Code '*Yes*' if the study quantitatively examined weekend effect on patient satisfaction and '*No*' if it did not.
**Patient satisfaction definition**<br>(free text)	Record how patient satisfaction was measured	e.g. what questionnaire was used or what/how the question was asked.
**Other outcomes of potential interest**	Record any other outcomes not listed above that were reported and might be useful	e.g. any process measures or costs information.<br>Record 'None' where appropriate.

**Variables/factors adjusted for** (free text)	List ALL variables that have been explored and/or included in the final multivariate model; or code as<br>• *None*	These could include:<br>Patient demographics and clinical conditions, such as age/age group, sex, race/ethnicity, insurance type, diagnosis/diagnosis-related group (DRG), comorbidity etc.<br>Physiological measures that reflect the severity/frailty/instability of patients' conditions, such as blood oxygen saturation, pulse rates and other blood biochemistry.<br>Provider characteristics, defined as features of health care organisations or health care professionals that could influence the capacity to provide high quality health care, such as hospital teaching status, hospital sizes, specialist centre designation, level of staffing (e.g. presence of consultants, nurse to patient ratio) and training or qualification of the doctors.<br>Other variables, such as measures of clinical processes (e.g. guideline adherence) or length of stay etc.
**Demographic – age** (*code*)	• *Yes*<br>• *No*<br>• *NA*	Code '*Yes'* if age or age group was adjusted in the multivariate analysis to estimate the weekend effect, *or* if the reported mortality rate was 'standardised' or 'matched' by age. Code '*No'* if it was not. Code '*NA*' if multivariate analysis was not performed.
**Demographic – sex** (*code*)	• *Yes*<br>• *No*<br>• *NA*	Code '*Yes'* if sex/gender was adjusted in the multivariate analysis to estimate the weekend effect, *or* if the mortality rate was 'standardised' or 'matched' by sex/gender. Code '*No'* if it was not. Code '*NA*' if multivariate analysis was not performed.
**Demographic – race/ethnicity** (*code*)	• *Yes*<br>• *No*<br>• *NA*	Code '*Yes'* if race/ethnicity was adjusted in the multivariate analysis to estimate the weekend effect. Code '*No'* if it was not. Code '*NA*' if multivariate analysis was not performed.
**Demographic – deprivation** (*code*)	• *Yes*<br>• *No*<br>• *NA*	Code '*Yes'* if deprivation, a related index or other measure of socioeconomic status (e.g. insurance type, social class) was adjusted in the multivariate analysis to estimate the weekend effect. Code '*No'* if it was not. Code '*NA*' if multivariate analysis was not performed.
**Reserve – comorbidity** (*code*)	• *Yes*<br>• *No*<br>• *NA*	Code '*Yes'* if comorbidity such as Charlson comorbidity index was adjusted in the multivariate analysis to estimate the weekend effect. Code '*No'* if it was not. Code '*NA*' if multivariate analysis was not performed.

**Diagnosis or diagnostic group**<br>(*code*)	• *Yes*<br>• *No*<br>• *NA*	Code '*Yes'* if diagnosis, diagnosis-related group (DRG), types of surgery (e.g. appendectomy, hip replacement) or other 'risk groups' was adjusted in the multivariate analysis to estimate the weekend effect. Code '*No'* if it was not. Code '*NA*' if multivariate analysis was not performed.
**Acute physiology or related score (e.g. NEWS)**<br>(*code*)	• *Yes*<br>• *No*<br>• *NA*	Code '*Yes'* if some measures of the patient's acute physiology such as NEWS score, blood oxygen saturation, pulse rates or other blood biochemistry was adjusted in the multivariate analysis to estimate the weekend effect. Code '*No'* if it was not. Code '*NA*' if multivariate analysis was not performed.
**Hospital characteristics (e.g. teaching status, bed size)**<br>(*code*)	• *Yes*<br>• *No*<br>• *NA*	Code '*Yes'* if one or more hospital characteristics was adjusted in the multivariate analysis to estimate the weekend effect. Code '*No'* if it was not. Code '*NA*' if multivariate analysis was not performed.
**Treatment pathway – emergency/urgent vs elective**<br>(*code*)	• *Yes*<br>• *No*<br>• *NA*	Code '*Yes'* if the types of admission related to emergency/urgent vs elective admissions was adjusted in the multivariate analysis to estimate the weekend effect. Code '*No'* if it was not. Code '*NA*' if the study focused on ONLY ONE of the following: emergency admissions, elective admissions, deliveries (childbirths); or if multivariate analysis was not performed.
**Treatment pathway – medical vs surgical**<br>(*code*)	• *Yes*<br>• *No*<br>• *NA*	Code '*Yes'* if the types of admission related to medical vs surgical admissions was adjusted in the multivariate analysis to estimate the weekend effect. Code '*No'* if it was not. Code '*NA*' if the study focused on ONLY ONE of the following: medical admissions, surgical admissions, deliveries (childbirths); or if multivariate analysis was not performed.
**Route of admission**<br>(*code*)	• *Yes*<br>• *No*<br>• *NA*	Code '*Yes'* if the route of admission was adjusted in the multivariate analysis to estimate the weekend effect. Code '*No'* if the study included emergency admissions but the route of admission was not adjusted. Code '*NA*' if the route of admission was unlikely to be varied or important, e.g. for elective admissions and deliveries (childbirths); or if multivariate analysis was not performed.

**Process measures or mediating / intermediate variables adjusted for**<br>(free text)	List (if any) process measures or mediating variables that were adjusted in multivariate analysis	These could include variables such as delay in receiving treatment or surgery, experiencing a complication or adverse event etc. The purpose of including such variables in multivariate analysis is usually to demonstrate that the variable(s) in question contribute/lead to the final outcome (death). For example, if adjustment of 'experiencing a complication' diminishes the weekend mortality effect related to surgical admissions, then it could be inferred that higher mortality at weekends were "mediated" through higher risk of experiencing a complication among weekend admissions.
**Assessment of model fit & the results**<br>(free text)	Describe methods used to evaluate how the statistical model performs in terms of correctly predicting the outcome and the results	This could be described as area under the receiver-operating characteristics (ROC) curve, c statistics etc.
**Odds ratios reported for all variables (i.e. not just weekend vs weekday) included in multivariate model**<br>(*code*)	• *Yes*<br>• *No*<br>• *NA*	In multivariate analysis (e.g. logistic regression), an odds ratio (or other effect measures) should ideally be reported for every variable included in the model so that we know whether and how much these variables can influence the outcome of interest (mortality following admissions). Code 'no' if the paper only reported the odd ratio related to weekend vs weekday admissions (the main explanatory variable of interest) but did not report odds ratios for other explanatory variables included in the model (e.g. age group, comorbidity etc.). Code '*Yes*'. if a table is provided which shows odds ratios for all variables included in the model. Code '*NA*' if multivariate analysis was not performed.
**Significant predictors from multivariate analysis (please list)**<br>(free text)	List all variables (other than weekday vs weekend admissions) which were found to be statistically significant in the multivariate analysis	The information (if available) can usually be found in a table that shows the results for multivariate analysis.<br>Just list the name of the variable – no need to record the numerical data at this stage.
**Risk of Bias Assessment**	The items below are modified from the Newcastle-Ottawa quality assessment scale	
**Selection**		

**Representativeness* of the weekend admissions**	Select ONE option from a) to d)	a) truly representative of the average weekend admissions<br>b) somewhat representative of the average weekend admissions<br>c) selected admissions<br>d) no description of the derivation of the cohort
**Selection of the weekday admissions**	Select ONE option from a) to c)	a) drawn from the same source as the weekend admissions<br>b) drawn from a different source<br>c) no description
**Ascertainment of admission day/time**	Select ONE option from a) to d)	a) secure record (e.g. hospital records)<br>b) structured interview<br>c) written self report<br>d) no description
**Demonstration that outcome of interest was not present at start of study**	Select a) or b)	a) yes<br>b) no
* This should be interpreted as “representative” of the average weekend admissions within the main scope of individual studies. For example if a study focused on all emergency admissions, we make a judgment on whether the admissions included in the study were representative of the average emergency admissions based on the stated inclusion/exclusion criteria. Similarly, for a study focused on elective surgical admissions, we make a judgement on whether the admissions included in the study were representative of the average elective surgical admissions. Please note this item focuses on representativeness in relation to stated inclusion/exclusion criteria other than exclusion due to missing data, which is now assessed in the last item under Outcome below.		
**Comparability**		

**Comparability of the cohorts on the basis of the (design or) analysis**	As patients admitted during weekends are likely to be different from those admitted during weekdays, we focus on adequacy of statistical adjustment here.<br><br>Select ONE option from 1) to 4)	**1) Comprehensive adjustment:**<br>Study adjusted for bot acute physiology and contextual factors listed below, as well as other important patient factors and treatment pathway<br>**2a) adequate adjustment – acute physiology**: study adjusted for acute physiology (includes early warning scores or other measures of severity of illness which include physiology) with or without adjusting for other major factors listed in 3) and 4)<br>**2b) adequate adjustment – contextual factors**: study adjusted for route of emergency admission (where applicable), i.e. through A & E (ambulance/999 or self-referral) vs through 'direct admission' (referral by outpatient clinic or GP) in addition to major factors listed in 3) below, but did not adjust for acute physiology<br>**3) partial adjustment**: study adjusted for important patient factors including age, main diagnosis, comorbidity/frailty indices AND treatment pathway (elective vs urgent/ emergency, operative vs non-operative) but did not adjust for factors listed in 2a) and 2b) above<br>**4) inadequate adjustment**: study did not adjust for some important factor(s) listed in 3) above or did not control for any factor at all
**Outcome**		
Assessment of outcome	Select ONE option from a) to d)	a) independent blind assessment<br>b) record linkage (e.g. information obtained from hospital records)<br>c) self report<br>d) no description
**Was follow up of outcomes beyond hospital stay?**	Select ONE option from a) to b)	a) yes<br>b) no
Exclusion due to missing data	Select ONE option from a) to e)	a) no or very few of exclusions due to missing data – unlikely to affect study results<br>b) some level of missing data, but admissions with missing data were retained in analyses using imputed data;<br>c) some level of exclusion due to missing data, but authors demonstrated that admissions with missing data were similar to admissions included in analyses<br>d) excluded a substantial proportion (5%) of admissions due to missing data from analyses<br>e) no statement concerning missing data
**Results**		

**Characteristics compared between weekday and weekend admissions** (free text)	Record variables which have been compared between weekday and weekend admissions.	These can usually be found in a table or in the first couple of paragraphs in the Results section.
**Significant differences observed in characteristics between weekday and weekend admissions** (free text)	Record the characteristics for which weekend admissions were found out to be significantly different from weekday admissions	List the names of the variables for which significant differences between weekday and weekend admissions were found. This can be defined statistically (i.e. $p<0.05$) or numerically (i.e. ≥5% difference between weekday/weekend admissions). No need to record numerical results at this stage.<br><br>For studies with a large sample size, trivial differences between weekday and weekend admission can still be statistically significant. Please add comments to describe if this is the case.
**Quantitative results**	These can be classified into two groups according to the types of outcome:<br>➢ Dichotomous (binary) variables such as deaths or occurrence of complications<br>➢ Continuous variables such as length of hospital stay	
**Results - dichotomous (binary) variables, e.g. death, complications** (free text)	Describe the features of the comparison being made; use one row for each set of data	Follow the format: [definition of outcome], [nature of admission: elective, emergency, maternity], [procedure involved: medical, surgical, childbirth], [type of patients: adult, paediatric, maternity], [comparison: weekends vs weekdays, out-of-hours vs regular hours], [time period, e.g. 2003-2004], [any other features of the comparison]. Omit the [item] if not relevant.<br>e.g. 7-day mortality, weekend vs weekday, 2008-2009 or<br>in-hospital mortality, emergency admissions, out of hours vs regular hours
**Number of events at weekends** (number)	Record the number of events (e.g. death) at weekends	This is the numerator for weekends
**Number of admissions at weekends** (number)	Record the total number of admissions at weekends	This is the denominator for weekends
**Event rate for weekend admissions**	Number of events divided by number of weekend admissions	Only record this if reported by the authors – no need to attempt calculation at this stage. Please clearly state the unit as it can vary, e.g. % or event per 1000 admissions

**Number of events on weekdays** (number)	Record the number of events (e.g. death) at weekdays	This is the numerator for weekdays
**Number of admissions on weekdays** (number)	Record the total number of admissions at weekdays	This is the denominator for weekdays
**Event rate for weekday admissions**	Number of events divided by number of weekday admissions	Only record this if reported by the authors – no need to attempt calculation at this stage. Please clearly state the unit as it can vary, e.g. % or event per 1000 admissions
**Unadjusted odds ratio (OR) & confidence interval**	May also be reported as relative risk (RR) or hazard ratio (HR)	Indicate using 'comment' if the measure is not OR; also make sure the comparison is weekends vs weekdays (i.e. weekday is the reference group)
**Adjusted odds ratio (OR) and confidence interval**	May also be reported as relative risk (RR) or hazard ratio (HR)	Indicate using 'comment' if the measure is not OR; also make sure the comparison is weekends vs weekdays (i.e. weekday is the reference group)
**Results - continuous variables, e.g. length of stay** (free text)	Describe the features of the comparison being made; use one row for each set of data	Follow the format: [definition of outcome], [nature of admission: elective, emergency, maternity], [procedure involved: medical, surgical, childbirth], [type of patients: adult, paediatric, maternity], [comparison: weekends vs weekdays, out-of-hours vs regular hours], [time period, e.g. 2003-2004], [any other features of the comparison]. Omit the [item] if not relevant.<br>e.g. Length of stay, weekend vs weekday, 2008-2009 or<br>patient satisfaction score, emergency admissions, out of hours vs regular hours
**Number of admissions on weekends** (number)	Record the number of weekday admissions contribute to this outcome	The number of weekend admissions included in the analysis can sometimes vary from outcome to outcome. Record NR if not reported
**Mean for weekend admissions**	Describe the mean value	Mean is the 'average' value.<br>Record 'NR' or 'NA' where appropriate
**Standard deviation (SD) or standard error (SE) for weekend admissions**	Describe the SD or SE value where reported	SD is a measure of how widely spread the values are surrounding the mean. SE is related to SD but is also influenced by the sample size. It is important to make sure whether the reported value is SD or SE.
**Median for weekend admissions**	Record the median value where reported	Median is the 'middle' value.<br>Record 'NR' or 'NA' where appropriate
**Interquartile range (IQR) for weekend admissions**	Record the IQR value where reported	IQR is the difference between the 25th percentile and 75th percentile. Sometimes these two values (rather than the difference between then) are provided. e.g. IQR 5 days or IQR 2 to 7 days.<br>Record 'NR' or 'NA' where appropriate

**Number of admissions on weekdays** (number)	Record the number of weekday admissions contribute to this outcome	The number of weekday admissions included in the analysis can sometimes vary from outcome to outcome. Record NR if not reported
**Mean for weekday admissions**	Describe the mean value	Mean is the 'average' value.<br>Record 'NR' or 'NA' where appropriate
**Standard deviation (SD) or standard error (SE) for weekday admissions**	Describe the SD or SE value where reported	SD is a measure of how widely spread the values are surrounding the mean. SE is related to SD but is also influenced by the sample size. It is important to make sure whether the reported value is SD or SE.
**Median for weekday admissions**	Record the median value where reported	Median is the 'middle' value.<br>Record 'NR' or 'NA' where appropriate
**Interquartile range (IQR) for weekday admissions**	Record the IQR value where reported	IQR is the difference between the 25th percentile and 75th percentile. Sometimes these two values (rather than the difference between then) are provided. e.g. IQR 5 days or IQR 2 to 7 days.<br>Record 'NR' or 'NA' where appropriate
**Difference between weekend and weekday admissions**	Record the difference between weekend and weekday admission for the continuous outcome	Could be reported as mean (SD) or median (IQR)<br>Record 'NR' if not reported

Appendix 2. Risk of bias assessment

Risk of bias assessment was embedded within the data extraction form shown in Appendix 1. We initially used the Newcastle-Ottawa scale [1] with modification of some of the items and wording because the included studies were mostly population database studies rather than the conventional cohort study for which the scale was designed.

However during the review process it became apparent that results of the risk of bias assessment using this modified scale were either unreliable (due to difficulties in judging the "representativeness" of the study sample for diverse types of admissions and lack of reported information about handling of missing data) or uninformative (e.g. all the included studies derived their control group [weekday admissions] from the same source and using the same inclusion criteria as with the exposure group [weekend admissions]). Therefore we subsequently only focus on adequacy of statistical adjustment, which was the key item stated *a priori* in our protocol.[2] The classification of statistical adjustment stated in the protocol needed to be refined during the review in view of emerging evidence indicating the importance of including measures of severity and urgency of the patients in the adjustment.

Discrepancies between reviewers in the classification were resolved by discussions between reviewers, and where queries remained, other review team members were supplied with information concerning statistical adjustment made in individual studies in the absence of study identity and outcome data to reach consensus prior to data analysis.

Appendix 3. Rationale and technical details of Bayesian analyses

3.1 Rationale for undertaking Bayesian meta-analysis and meta-regression

Bayesian methods for meta-analysis offer several advantages over alternatives as they permit the development of more flexible, multiple-level hierarchical models, make full allowance for uncertainty in hierarchical model parameters, and have a more intuitive interpretation of the results.[3]

3.2 Technical details of the Bayesian meta-analysis

Analyses were undertaken using (log) adjusted odds ratios. For studies that only reported adjusted hazard ratios or rate ratios, we used these figures as approximations of adjusted odds ratios as results for these effect measures were very similar where they had been estimated in the same study (see Appendix 7.3.3).

As several studies provided multiple estimates of the weekend effect from different sub-samples (e.g. different time periods or different locations), we specified a three level Bayesian random-effects model to take into account the correlation of results from different sub-samples within the same study while allowing for within sample variation and between study heterogeneity. In particular, for analysis or sub-sample $n = 1, \dots, N$ from study $j = 1, \dots, J$ with effect size estimate y_{nj} and estimated standard deviation s_{nj}:

$$
\begin{aligned}
y_{nj} &\sim N\left(\theta_{nj}, s_{nj}^2\right) \\
\theta_{nj} &\sim N\left(\alpha_j, \sigma^2\right) \qquad (1) \\
\alpha_j &\sim N(\mu, \tau^2)
\end{aligned}
$$

$$\sigma^2 \sim N(0,1)I[0,\infty), \tau^2 \sim N(0,1)I[0,\infty), \mu \sim N(0,5^2)$$

Weakly informative priors were specified for the model parameters in order to constrain the parameter to realistic values and provide a degree of regularisation (which facilitates computation especially with small numbers of studies) while providing relatively little information to influence the posterior estimates.[4] For example, a N(0,1)I[0,] prior for between study heterogeneity has a 95th percentile of 1.96, which would be considered large given a within study estimated standard deviation for the weekend effect of between approximately 0.01 and 0.05. Previous research also suggests higher level variance terms in meta-analysis rarely exceed 0.2 in these contexts (see Turner et al. 2015).[5] We therefore used half-normal(0,1) priors for standard deviation terms and normal(0,1) for mean effects. We calculated the I-squared statistic,[6] which is the proportion of total variance attributable to between-study heterogeneity taking into account variance at three levels. Convergence was assessed by visual inspection of traceplots of MCMC chains and the Rhat statistic. Models were estimated in Stan.[7]

3.3 Technical details of the Bayesian meta-regression

The model described in Equation (1) is extended to allow for varying mean effects according to characteristics of the sample, x_{nj}:

$$y_{nj} \sim N\left(\theta_{nj} + x'_{nj}\beta, s^2_{nj}\right)$$

where β are a set of parameters to be estimated.

The following variables were included in a planned, exploratory meta-regression:

- Binary variable indicating whether the study data contained records of emergency admissions
- Binary variable indicating whether the study data included records of surgical patients
- A linear time trend. Where there were multiple years of data in the study, the mid-point was used.
- Categorical variable indicating adequacy of case-mix adjustment as described earlier. Reference category was combined 1 and 2a (with adjustment of measures of acute physiology).

Two pre-specified variables were not included in the meta-regression due to lack of data: type of population (few studies focused on children) and country income category (none of the included studies was conducted in low and middle income countries). Instead we included an indicator variable for each country. The country effect is specified as a hierarchical ‘random’ effect.

Appendix 4. Examination of potential overlap in the coverage of admissions between different studies

Many studies included in this systematic review utilised data from routine administrative databases, most prominently the Hospital Episode Statistics (HES) from England and the National Inpatient Sample (NIS) from USA. Inclusion of studies that cover data related to the same or overlapping admissions in a meta-analysis results in double-counting and therefore needs to be avoided.

In the tables below we summarise characteristics of studies based in England and USA and illustrate the extent of potential overlap of data between these studies. Attention was paid to the hierarchical nature of the data; for example a study that included all emergency admissions would have included the same data from another study that focused on emergency medical admissions if they used the same data source and covered the same period of time, even though the former may not have provided an estimate of the weekend effect specific to emergency medical admissions.

For meta-regression, we included the most relevant estimate(s) from individual studies irrespective of whether their data overlaps with each other, as the main purpose is to explore factors that may influence the estimated magnitude of the weekend effect rather than to provide a summary estimate across studies.

4.1 Potential overlap in data between studies of hospital mortality in England

Table 1 List of studies examining mortality outcomes that were based on data from English hospitals

Study	Statistical adjustment	Location	Data source	Study period	Type of admissions
McCallum 2016 [8]	2b	England	HES	2000-2014	Emergency surgical
Roberts 2015 [9]	4	England	HES	2004-2012	Emergency (both medical & surgical)
Han 2017 [10]	2b	Salford (1 hospital)	Hospital patient record	2004-2014	Emergency (both medical & surgical)
Aylin 2010 [11]	3	England	NHS Wide Clearing Service	2005-2006	Emergency (both medical & surgical)
Ozdemir 2016 [12]	3	England	HES	2005-2010	Emergency surgical
Walker 2017 [13]	1	Oxford (4 hospitals)	Infections in Oxfordshire Research Database	2006-2014	Emergency (both medical & surgical)
Maggs 2010 [14]	4	Bath (1 hospital)	Anonymised hospital activity data	2007-2008	Emergency medical
Mohammed 2012 [15]	3	England	HES	2008-2009	Emergency & elective (both medical & surgical)
Aylin 2013 [16]	2b	England	HES	2008-2011	Elective surgical
Ruiz 2016 [17]	2b	England	HES	2008-2011	Elective surgical
Freemantle 2012 [18]	2b	England	HES	2009-2010	All admissions (including maternity)
Ruiz 2015 [19]	3	England (11 hospitals)	HES	2009-2012	Emergency (both medical & surgical) and elective surgical
Meacock 2015 [20]	4	England	HES	2010-2011	Emergency (both medical & surgical)
Palmer 2015 [21]	2b	England	HES	2010-2012	Maternity admissions
Shiue 2017 [22]	4	Newcastle (1 hospital)	HES	2010-2015	Emergency (both medical & surgical)
Freemantle 2015 [23]	2b	England	HES	2013-2014	All admissions
Aldridge 2016 [24]	3	England	HES	2013-2014	Emergency (both medical & surgical)
Anselmi 2016 [25]	2b	England	HES	2013-2014	Emergency (both medical & surgical)
Meacock 2016 [26]	2b	England	HES	2013-2014	Emergency (both medical & surgical)
Meacock 2017 [27]	2b	England	HES	2013-2016	Emergency admissions & all admissions
Mohammed 2017 [28]	2a	Yorkshire & Humberside (4 hospitals)	Hospital database	2014	Emergency medical

Statistical adjustment: 1) Comprehensive adjustment; 2) Adequate adjustment: 2a – adjusted for measures of acute physiology; 2b – adjusted for contextual factors reflecting the severity or urgency of the patient's condition, such as

whether the patient was admitted through A & E and/or brought in by ambulance; 3) Partial adjustment; and 4) Inadequate adjustment

HES: Hospital Episode Statistics

Table 2 Potential overlap between studies based on data from English hospitals

00-04	04-05	05-06	06-07	07-08	08-09	09-10	10-11	11-12	12-13	13-14	14-15	15-16
**1. All admissions**												
					Mohammed 2012	Freemantle 2012				Freemantle 2015		
						Ruiz 2015*	Ruiz 2015*	Ruiz 2015*				
										Meacock 2017	Meacock 2017	Meacock 2017
**1.1 All medical admissions**												
					Mohammed 2012	Freemantle 2012				Freemantle 2015		
										Meacock 2017	Meacock 2017	Meacock 2017
**1.2 All surgical admissions**												
					Mohammed 2012	Freemantle 2012				Freemantle 2015		
						Ruiz 2015*	Ruiz 2015*	(Ruiz 2015*		Meacock 2017	Meacock 2017	Meacock 2017
**1.3 All emergency admissions**												
		Aylin 2010			Mohammed 2012	Freemantle 2012	Meacock 2015			Aldridge 2016		
	Roberts 2015	Roberts 2015	Roberts 2015	Roberts 2015	Roberts 2015	Roberts 2015	Roberts 2015	Roberts 2015		Anselmi 2016		
	Han 2017*	Han 2017*	Han 2017*	Han 2017*	Han 2017*	Han 2017*	Han 2017*	Han 2017*	Han 2017*	Han 2017*		
										Freemantle 2015		
			Walker 2017*	Walker 2017*	Walker 2017*	Walker 2017*	Walker 2017*	Walker 2017*	Walker 2017*	Walker 2017*		
						Ruiz 2015*	Ruiz 2015*	Ruiz 2015*		Meacock 2016		
							Shiue 2017*	Shiue 2017*	Shiue 2017*	Shiue 2017*	Shiue 2017*	
										Meacock 2017	Meacock 2017	Meacock 2017
**1.3.1 Emergency medical admissions**												
	Roberts 2015	Roberts 2015	Roberts 2015	Roberts 2015	Roberts 2015	Roberts 2015	Roberts 2015	Roberts 2015		Aldridge 2016		
		Aylin 2010			Mohammed 2012	Freemantle 2012	Meacock 2015			Anselmi 2016		
						Ruiz 2015*	Ruiz 2015*	Ruiz 2015*		Freemantle 2015		

			Walker 2017*	Walker 2017*	Walker 2017*	Walker 2017*	Walker 2017*	Walker 2017*	Walker 2017*	Walker 2017*		
										Meacock 2016		
										Meacock 2017	Meacock 2017	Meacock 2017
				Maggs 2010*						Mohammed 2017*	Mohammed 2017* (year 2014)	
**1.3.2 Emergency surgical admissions**												
McCallum 2016	McCallum 2016	McCallum 2016	McCallum 2016	McCallum 2016	McCallum 2016	McCallum 2016	McCallum 2016	McCallum 2016	McCallum 2016	McCallum 2016		
	Roberts 2015	Roberts 2015	Roberts 2015	Roberts 2015	Roberts 2015	Roberts 2015	Roberts 2015	Roberts 2015		Aldridge 2016		
		Ozdemir 2016	Ozdemir 2016	Ozdemir 2016	Ozdemir 2016	Ozdemir 2016	Meacock 2015			Anselmi 2016		
		Aylin 2010			Mohammed 2012	Freemantle 2012				Freemantle 2015		
			Walker 2017*	Walker 2017*	Walker 2017*	Walker 2017*	Walker 2017*	Walker 2017*	Walker 2017*	Walker 2017*		
										Meacock 2016		
						Ruiz 2015*	Ruiz 2015*	Ruiz 2015*		Meacock 2017	Meacock 2017	Meacock 2017
**1.4 All elective admissions**												
					Mohammed 2012	Freemantle 2012 (non-emergency)				Freemantle 2015		
										Meacock 2017	Meacock 2017	Meacock 2017
**1.4.1 Elective surgical admissions**												
					Aylin 2013	Aylin 2013	Aylin 2013					
					Ruiz 2016	Ruiz 2016	Ruiz 2016					
					Mohammed 2012	Freemantle 2012				Freemantle 2015		
						Ruiz 2015*	Ruiz 2015*	Ruiz 2015*		Meacock 2017	Meacock 2017	Meacock 2017
**1.5 Maternity admissions**												
						Freemantle 2012	Palmer 2015	Palmer 2015		Freemantle 2015		

(grey)	Data available only at a higher level (aggregated with other sub-types of admissions)	(yellow)	Best adjusted, minimally overlapping data for each sub-type of admission

* indicates non-database studies, i.e. the studies were carried out in a small number of hospitals

4.2 Potential overlap in data between studies of hospital mortality in the USA

Table 3 List of studies examining mortality outcomes that were based on data from US hospitals

	**Statistical adjustment**	**Sampling location [number of hospitals]**	**Population**	**Study period**	**Type of admissions**
Goldstein 2014 [29]	2b	Nationwide [NR]	Children only	1988-2010	Emergency surgical
Gordon 2005 [30]	4	VA hospitals [44]	Adults	1991-1993	All non-cardiac surgical (both emergency & elective)
Gould 2003 [31]	3	California [NR]	Maternity	1995-1997	Maternity
Cram 2004 [32]	3 (2b for stratified analysis)	California [NR]	Adults only	1998	All admissions (excluding maternity)
Hamilton 2006 [33]	4	Texas [NR]	Maternity	1999-2001	Maternity
Zare 2007 [34]	2b	VA hospitals [124]	Adults only	2000-2004	Elective surgical
Attenello 2015 [35]	2b	Nationwide [NR]	All patients	2002-2010	All admissions
Ricciardi 2011 [36]	3	Nationwide [1000]	All patients	2003-2007	Non-elective (both medical & surgical)
Ricciardi 2014 [37]	3	Nationwide [1000]	All patients	2003-2008	Non-elective (both medical & surgical)
Ricciardi 2016 [38]	4	Nationwide [1000]	All patients	2003-2008	Non-elective (both medical & surgical)
An 2017 [39]	3	Nationwide [1000]	Adults only	2003-2013	Non-elective (both medical & surgical)
Zapf 2015 [40]	3	Florida [NR]	All patients	2007-2010	Emergency surgical
Sharp 2013 [41]	4	Nationwide [NR]	Adults only	2008	Emergency (both medical & surgical)
Snowden 2016 [42]	4	California [214]	Maternity	2009-2010	Maternity

Statistical adjustment: 1) Comprehensive adjustment; 2) Adequate adjustment: 2a – adjusted for measures of acute physiology; 2b – adjusted for contextual factors reflecting the severity or urgency of the patient's condition, such as whether the patient was admitted through A & E and/or brought in by ambulance; 3) Partial adjustment; and 4) Inadequate adjustment. NR: not reported; VA: Veterans Affairs

Table 4 Potential overlap between studies based on data from US hospitals

	88-91	91-93	93-97	98	99-02	02-03	03-04	04-05	05-06	06-07	07-08	08-09	09-10	11-12	12-13
**1. All admissions**															
Nationwide (NIS) all patients						Attenello 2015	Attenello 2015	Attenello 2015	Attenello 2015	Attenello 2015	Attenello 2015	Attenello 2015	Attenello 2015		
California				Cram 2004											
**Global comparator project**													(Ruiz 2015)	(Ruiz 2015)	
**1.1 All medical admissions**															
Nationwide (NIS) all patients						Attenello 2015	Attenello 2015	Attenello 2015	Attenello 2015	Attenello 2015	Attenello 2015	Attenello 2015	Attenello 2015		
				Cram 2004											
**1.2 All surgical admissions**															
Nationwide (NIS) all patients						Attenello 2015	Attenello 2015	Attenello 2015	Attenello 2015	Attenello 2015	Attenello 2015	Attenello 2015	Attenello 2015		
VA hospitals, non-cardiac		Gordon 2005													
California				Cram 2004											
**Global comparator project**													(Ruiz 2015)	(Ruiz 2015)	
**1.3 All emergency admissions**															
Nationwide (NIS) all patients						Attenello 2015	Attenello 2015	Attenello 2015	Attenello 2015	Attenello 2015	Attenello 2015	Attenello 2015	Attenello 2015		
Nationwide (NIS) all patients							Ricciardi 2011	Ricciardi 2011	Ricciardi 2011	Ricciardi 2011					
Nationwide (NIS) all patients							Ricciardi 2014	Ricciardi 2014	Ricciardi 2014	Ricciardi 2014	Ricciardi 2014				
Nationwide (NIS) all patients							Ricciardi 2016	Ricciardi 2016	Ricciardi 2016	Ricciardi 2016	Ricciardi 2016				
Nationwide (NIS) adults only							An 2017	An 2017	An 2017	An 2017	An 2017	An 2017	An 2017	An 2017	An 2017
Nationwide (NEDS) adults, through ED											Sharp 2013 (year 2008)				
California				Cram 2004 (unscheduled admissions)											
**Global comparator project**													(Ruiz 2015)	(Ruiz 2015)	

**1.3.1 Emergency medical admissions**															
Nationwide (NIS) all patients						Attenello 2015	Attenello 2015	Attenello 2015	Attenello 2015	Attenello 2015	Attenello 2015	Attenello 2015	Attenello 2015		
Nationwide (NIS) all patients							Ricciardi 2011	Ricciardi 2011	Ricciardi 2011	Ricciardi 2011					
Nationwide (NIS) all patients							Ricciardi 2014	Ricciardi 2014	Ricciardi 2014	Ricciardi 2014	Ricciardi 2014				
Nationwide (NIS) all patients							Ricciardi 2016	Ricciardi 2016	Ricciardi 2016	Ricciardi 2016	Ricciardi 2016				
Nationwide (NEDS) adults, through ED											Sharp 2013 (year 2008)				
California				Cram 2004											
**Global comparator project**													(Ruiz 2015)	(Ruiz 2015)	
**1.3.2 Emergency surgical admissions**															
Nationwide (NIS) all patients						Attenello 2015	Attenello 2015	Attenello 2015	Attenello 2015	Attenello 2015	Attenello 2015	Attenello 2015	Attenello 2015		
Nationwide (NIS) all patients							Ricciardi 2011	Ricciardi 2011	Ricciardi 2011	Ricciardi 2011					
Nationwide (NIS) all patients							Ricciardi 2014	Ricciardi 2014	Ricciardi 2014	Ricciardi 2014	Ricciardi 2014				
Nationwide (NIS) all patients							Ricciardi 2016	Ricciardi 2016	Ricciardi 2016	Ricciardi 2016	Ricciardi 2016				
Nationwide (NEDS) adults, through ED											Sharp 2013 (year 2008)				
Nationwide (NIS & KID) children	Goldstein 2014	Goldstein 2014	Goldstein 2014	Goldstein 2014	Goldstein 2014	Goldstein 2014	Goldstein 2014	Goldstein 2014	Goldstein 2014	Goldstein 2014	Goldstein 2014	Goldstein 2014	Goldstein 2014		
VA hospitals, non-cardiac		Gordon 2005													
California				Cram 2004											
Florida											Zapf 2015	Zapf 2015	Zapf 2015		
**Global comparator project**													(Ruiz 2015)	(Ruiz 2015)	
**1.4 All elective admissions**															
Nationwide (NIS) all patients						Attenello 2015	Attenello 2015	Attenello 2015	Attenello 2015	Attenello 2015	Attenello 2015	Attenello 2015	Attenello 2015		
**1.4.1 Elective medical**															
Nationwide (NIS) all patients						Attenello 2015	Attenello 2015	Attenello 2015	Attenello 2015	Attenello 2015	Attenello 2015	Attenello 2015	Attenello 2015		
California				Cram 2004											

**1.4.2 Elective surgical**															
Nationwide (NIS) all patients						Attenello 2015	Attenello 2015	Attenello 2015	Attenello 2015	Attenello 2015	Attenello 2015	Attenello 2015	Attenello 2015		
VA hospitals, non-cardiac		Gordon 2005				Zare 2007	Zare 2007								
California				Cram 2004											
**Global comparator project**													(Ruiz 2015)	(Ruiz 2015)	
**1.5 Maternity**															
Nationwide (NIS) all patients						Attenello 2015	Attenello 2015	Attenello 2015	Attenello 2015	Attenello 2015	Attenello 2015	Attenello 2015	Attenello 2015		
California													Snowden 2016		
Texas					Hamilton 2006										

	Data available only at a higher level (aggregated with other sub-type of admissions)		Best adjusted, minimally overlapping data for each sub-type of admission

* indicates non-database studies, i.e. the studies were carried out in a small number of hospitals

ED: emergency department; KID: Kids' Inpatient Database; NEDS: Nationwide Emergency Department Sample; NIS: National (Nationwide) Inpatient Sample.

Appendix 5. PRISMA flow diagram

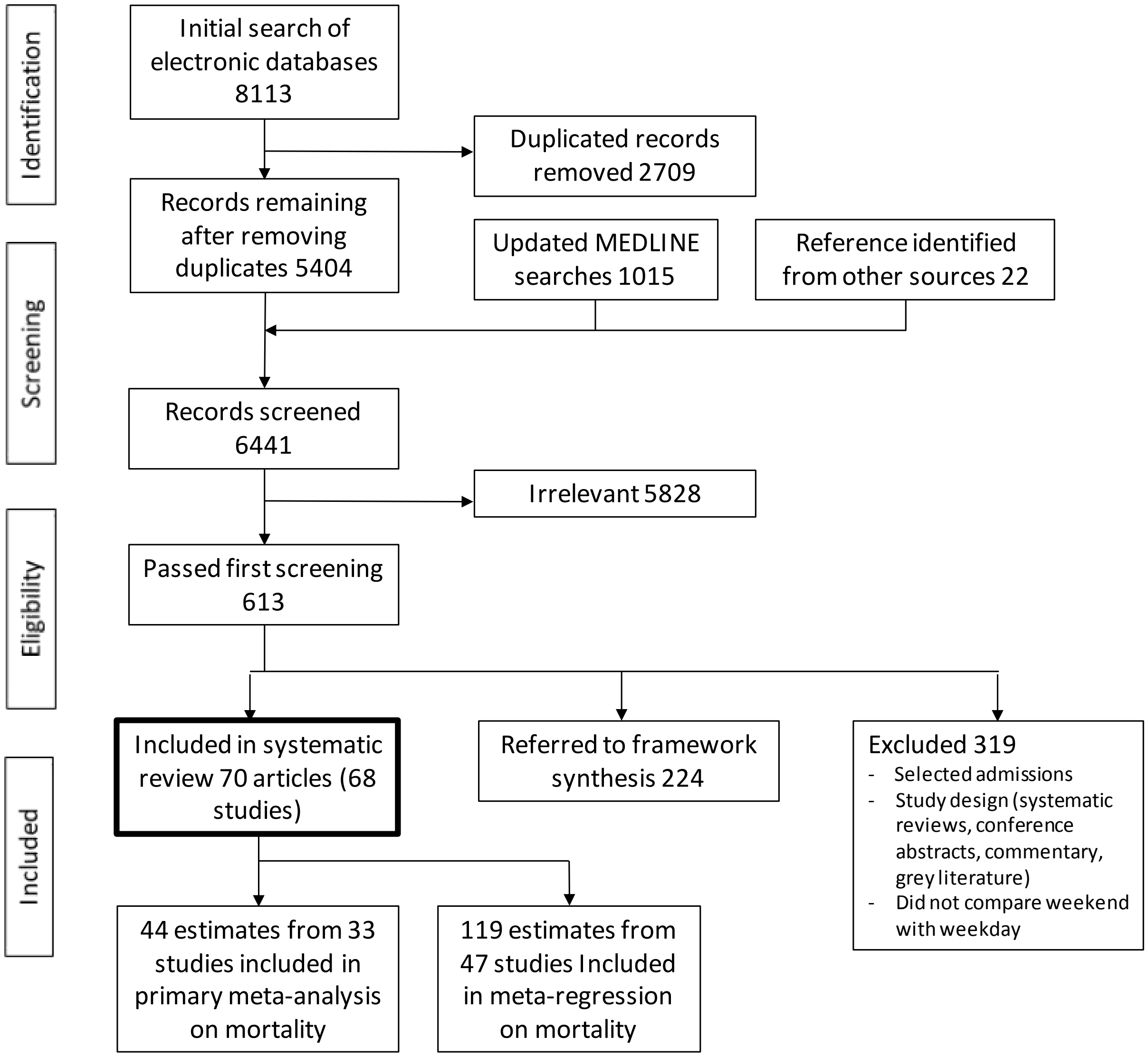

Figure 1 PRISMA flow diagram for literature search, study screening and selection

Appendix 6. Characteristics of included studies (sorted by type of admissions)

Table 5 Characteristics of studies included in this review

Study [rating of statistical adjustment][$]	Country [number of hospitals]	Study period	Sample size (admissions unless otherwise stated)	Type of admission (emergency, elective)	Type of procedure (medical, surgical, maternity)	Population	Mortality outcomes	Other outcomes: Adverse events Length of stay Patient satisfaction
**All admissions (including both medical and surgical, emergency and elective admissions)**								
Attenello 2015 [35] [3]	USA (nationwide) [NR]	2002 to 2010	351170803	Emergency & elective	Medical & surgical	All patients	In-hospital	Hospital acquired conditions
Auger 2015 [43] [4]	USA (Michigan) [1]	2006 to 2012	55383	Emergency & elective	Medical & surgical	Children only	-	Unplanned readmission
Coiera 2014 [44] [3]	Australia (New South Wales) [501]	2000 to 2007	11732260	Emergency & elective	Medical, surgical & maternity	All patients	In/out hospital up to 7 days post-discharge*	-
Cram 2004 [32] [2b or 3][a]	USA (California) [NR]	1998	1100984	Emergency & elective	Medical & surgical	Adults only	In-hospital	-
Earnest 2006 [45] [4]	Singapore [1]	2003 to 2004	45395	Emergency & elective	Medical & surgical	Adults only	-	Length of stay
Freemantle 2012 [18] [2b]	UK (England) [NR] & USA (United Health Care System) [254]	2009 to 2010	14217640	Emergency & elective	Medical, surgical & maternity	All patients	In/out hospital (30-day); also in-hospital (30-day) and 3-day	-
Freemantle 2015 [23] [2b]	UK (England) [NR]	2013 to 2014	14818374	Emergency & elective	Medical, surgical & maternity	All patients	In/out hospital (30-day)	Length of stay
Graham 2017 [46] [4]	UK (England) (NR)	2014	59083	Emergency & elective	Medical & surgical	Adults only	-	Patient satisfaction
Lee 2012 [47] [4]	Malaysia (Perak) [1]	2008 to 2010	126627	Emergency & elective	Medical & surgical	All patients	In-hospital	-
Mohammed 2012 [15] [3]	UK (England) [NR]	2008 to 2009	4640516	Emergency & elective	Medical & surgical	Adults only	In-hospital (at discharge)	
Ruiz 2015 [19] [3]	International: UK (England) [11], Australia [6],	2009 to 2012	2982570	Emergency (all) & elective (surgical only)	Medical & surgical	All patients	In-hospital (30-day)	-

Study [rating of statistical adjustment][$]	Country [number of hospitals]	Study period	Sample size (admissions unless otherwise stated)	Type of admission (emergency, elective)	Type of procedure (medical, surgical, maternity)	Population	Mortality outcomes	Other outcomes: Adverse events Length of stay Patient satisfaction
	Netherlands [6], USA [5]							
**Medical admissions**								
Madsen 2014 [48] [4]	Denmark (nationwide) [72]	1995 to 2012	2651021	Emergency & elective	Medical	Adults only	In-hospital and 30-day*	-
**Surgical admissions**								
Bendavid 2007 [49] [3]	USA (New York, Massachusetts, North Carolina) [NR]	1999 to 2001	4967114	Emergency & elective	Surgical & obstetric	All patients	-	AHRQ Patient Safety Indicator (surgical & birth complications)
Gordon 2005 [30] [4]	USA (VA hospital) [44]	1991 to 1993	78546	Emergency & elective	Surgical (non-cardiac)	Adults	In-hospital (30-day)	-
Ozrazgat-Baslanti 2016 [50] [3]	USA (Florida) [1]	2000 to 2010	50314	Emergency & elective	Surgical	Adults only	-	Post-operative complications
**Emergency admissions: medical & surgical**								
Aldridge 2016 [24] [3]	UK (England) [141]	2013 to 2014	4,422,387	Emergency	Medical & surgical	Adults only	In-hospital	-
An 2017 [39] [3]	USA (nationwide) [NR]	2003 to 2013	51,762,178	Non-elective	Medical, surgical & maternity	Adults only	In-hospital	Length of stay
Anselmi 2016 [25] [2b]	UK (England) [140]	2013 to 2014	3,027,946	Emergency	Medical & surgical	Not stated	In-hospital (30-day)	-
Aylin 2010 [11] [3]	UK (England) [163]	2005 to 2006	4,317,866	Emergency	Medical & surgical	Adults & children	In-hospital	-
Barba 2006 [51] [3]	Spain (Madrid) [1]	1999 to 2003	35,993	Emergency	Medical & surgical	Adults only	In-hospital (2-day & any)	-
Bell 2001 [52] [4][b]	Canada (Ontario) [NR]	1988 to 1997	3,789,917	Emergency	Medical & surgical	Adults & children	In-hospital*	-
De Giorgi 2015 [53] [4]	Italy (Ferrara) [1]	2000 to 2013	411,588	Emergency	Medical & surgical	All patients	In-hospital (30-day)	-
Han 2017 [10] [2b]	UK (Salford) [1]	2004 to 2014	246,350	Emergency	Medical & surgical	Adults only	In-hospital, 7-day & 30-day	-
Handel 2012 [54] [4]	UK (Scotland) [NR]	1999 to 2009	5,271,327	Emergency	Medical & surgical	All patients	In-hospital	-

Study [rating of statistical adjustment][$]	Country [number of hospitals]	Study period	Sample size (admissions unless otherwise stated)	Type of admission (emergency, elective)	Type of procedure (medical, surgical, maternity)	Population	Mortality outcomes	Other outcomes: Adverse events Length of stay Patient satisfaction
Lee 2006 [55] [3]	Taiwan (nationwide) [NR]	2000 to 2002	712,787^	Emergency	Medical & surgical	Adults & children	In/out hospital (24-hour, 48-hour, 30-day)	-
Meacock 2015 [20] [4]	UK (England) [NR]	2010 to 2011	5,212,973	Emergency	Medical & surgical	Not stated	In/out hospital (30-day)*	-
Meacock 2016 [26] [2b]	UK (England) [140]	2013 to 2014	4,656,586	Emergency	Medical & surgical	All patients	In-hospital (30-day)	-
Meacock 2017 [27] [3]	UK (England) [123]	2013 to 2016	Not stated	Emergency (also included supplementary data on all admissions)	Medical & surgical	Not stated	In/out hospital (30-day)	-
Perez Concha 2014 [56] [3]	Australia (New South Wales) [501]	2000 to 2007	3381962	Emergency	Medical & surgical	All patients	In/out hospital (7-day); also reported in-hospital & post-discharge separately	-
Ricciardi 2011 [36] [3]	USA (nationwide) [1000]	2003 to 2007	29,991,621	Emergency	Medical & surgical	All patients	In-hospital (vital status at discharge)	-
Ricciardi 2014 [37] [3]	USA (nationwide) [1000]	2003 to 2008	26,051,775	Emergency	Medical & surgical	All patients	In-hospital (vital status at discharge)*	-
Ricciardi 2016 [38] [4]	USA (nationwide) [1000]	2003 to 2008	28,236,749	Emergency	Medical & surgical	All patients	In-hospital (vital status at discharge)	Patient safety indicators
Roberts 2015 [9] [4]	UK (England & Wales) [NR]	2004 to 2012	32,628,333	Emergency	Medical & surgical	Not stated	30-day (location not specified)	-
Sharp 2013 [41] [4]	USA (nationwide) [NR]	2008	4,225,973	Emergency	Medical & surgical	Adults only	Mortality (not specified)	-
Shiue 2017 [22] [4]	UK (Newcastle) [1]	2010 to 2015	148,996	Emergency	Medical & surgical	Not stated	In/out hospital (30-day)	-

Study [rating of statistical adjustment][$]	Country [number of hospitals]	Study period	Sample size (admissions unless otherwise stated)	Type of admission (emergency, elective)	Type of procedure (medical, surgical, maternity)	Population	Mortality outcomes	Other outcomes: Adverse events Length of stay Patient satisfaction
Sullivan 2016 [57] [4]	Australia (Queensland) [1]	2011 & 2013	34184	Emergency	Medical & surgical	All patients	In-hospital (timing not specified)	-
Walker 2017 [13] [1]	UK (Oxford) [4]	2006 to 2014	503,938	Emergency	Medical & surgical	All patients	In/out hospital (30-day)	Admission to ICU
**Emergency medical admissions**								
Conway 2016, [58] 2017a, [59] 2017b [60] [2a or 4][c]	Ireland (Dublin) [1]	2002 to 2014	82,368	Emergency	Medical	All patients	In-hospital (30-day)	-
Khanna 2011 [61] [2b]	USA (Chicago) [1]	2008	824	Emergency	Medical	All patients	-	Need for ICU, 30-day ED re-visit, 30-day readmission, poor outcomes in the first 24 hours
Maggs 2010 [14] [4]	UK (Bath) [1]	2007 to 2008	15,594	Emergency	Medical	Adults only	In-hospital and ‘late’ mortality (in-hospital death beyond the first 7 days)	-
Mikulich 2011 [62] [2a]	Ireland (Dublin) [1]	2002 to 2009	25,833	Emergency	Medical	Adults only	In-hospital (30-day)	Length of stay
Mohammed 2017 [28] [2a]	UK (Yorkshire & Humberside) [4]	2014	47,117	Emergency	Medical	Adults only	In-hospital (vital status at discharge)	Length of stay
Vest-Hansen 2015 [63] [4]	Denmark (nationwide) [NR]	2010	174,192	Emergency	Medical	Adults only	In/out hospital (30-day)	Length of stay
**Emergency surgical admissions**								
Beecher 2015 [64] [4]	Ireland (Galway) [1]	2012 to 2013	7,041	Emergency	Surgical	Not stated	-	Length of stay
Gillies 2017 [65] [3]	UK (Scotland) [NR]	2005 to 2007	50,844	Emergency	Surgical	Adults only	In-hospital or within 30 days; overall survival (4-years)	-

Study [rating of statistical adjustment]$	Country [number of hospitals]	Study period	Sample size (admissions unless otherwise stated)	Type of admission (emergency, elective)	Type of procedure (medical, surgical, maternity)	Population	Mortality outcomes	Other outcomes: Adverse events Length of stay Patient satisfaction
Goldstein 2014 [29] [2b]	USA (nationwide) [NR]	1988 to 2010	439,457	Emergency	Surgical	Children only	In-hospital	Various surgical complications; length of stay
McCallum 2016 [8] [2b]	UK (Northern England) [NR]	2000 to 2014	370,671	Emergency	Surgical	Adults only	In-hospital (30-day)	Length of stay
Ozdemir 2016 [12] [3]	UK (England) [156]	2005 to 2010	294,602	Emergency	General surgical	All patients	In/out hospital (30-day & 90-day)	-
Zapf 2015 [40] [3]	USA (Florida) [NR]	2007 to 2010	80,861	Emergency	Surgical	All patients	In-hospital (timing not specified)	Postoperative complications, length of stay
**Elective surgical admissions**								
Aylin 2013 [16] [2b]	UK (England) [163]	2008 to 2011	4,133,346	Elective	Surgical	Adults & children	In/out hospital, (30-day & 2-day)	-
Dubois 2016 [66] [2b]	Canada (Ontario) [NR]	2002 to 2012	402,899	Elective	Surgical (day of surgery)	Adults only	In/out hospital (30-days), also 2-day and 90-day and in-hospital	Admission to ICU; readmission (30-day); reoperation (30-day); length of stay
McIsaac 2014 [67] [2b]	Canada (Ontario) [NR]	2002 to 2012	333,344	Elective	Surgical (non-cardiac)	Adults only (≥40 years)	In/out hospital (30-day & 2-day)	-
Ruiz 2016 [17] [2b]	UK (England) [163]	2008 to 2011	3,922,091	Elective	Surgical	Adults & children	In/out hospital (30-day)	-
Zare 2007 [34] [2b]	USA (VA hospitals) [124]	2000 to 2004	188,212	Elective	Surgical	Adults only	30-day (location not specified)	Post-operative morbidity (complications)
**Maternity admissions**								
de Graaf 2010 [68] [2b]	Netherlands (nationwide) [99]	2000 to 2006	764,406	Spontaneous & elective	Medical & surgical	Maternity	Intrapartum & early neonatal (within 7 days of birth)	5-minute Apgar score < 7; transfer to neonatal ICU
Frank-Wolf 2016 [69] [4]	Israel [1]	2005 to 2014	56,428	Spontaneous & elective	Medical & surgical	Maternity	-	Cord blood pH <7; 5 minute Apgar score <7

Study [rating of statistical adjustment][$]	Country [number of hospitals]	Study period	Sample size (admissions unless otherwise stated)	Type of admission (emergency, elective)	Type of procedure (medical, surgical, maternity)	Population	Mortality outcomes	Other outcomes: Adverse events Length of stay Patient satisfaction
Gijsen 2012 [70] [2b]	Netherlands (nationwide) [NR]	2003 to 2007	449,714	Spontaneous	Medical & surgical	Maternity	Intrapartum & early neonatal (within 7 days of birth)	5-minute Apgar score < 7; a composite measure of adverse outcomes
Gould 2003 [31] [3]	USA (California) [NR]	1995 to 1997	1,615,041	Spontaneous & elective	Medical & surgical	Maternity	Neonatal mortality (within 28 days of birth)	-
Hamilton 2006 [33] [4]	USA (Texas) [NR]	1999 to 2001	923,905	Not stated	Not stated	Maternity	Neonatal mortality (within 27 days of birth)*	-
Luo 2004 [71] [4]	Canada (nationwide, excluding Ontario) [NR]	1985 to 1998	3,239,972	Spontaneous & elective	Medical & surgical	Maternity	Neonatal mortality (within 6 days of birth)	-
Lyndon 2015 [72] [4]	USA (California) [NR]	2005 to 2007	1,475,593	Spontaneous & elective	Medical & surgical	Maternity	-	Pelvic morbidity, severe maternal morbidity
Palmer 2015 [21] [2b]	UK (England) [NR]	2010 to 2012	1,332,835 maternity admissions and 1,349,599 births	Spontaneous & elective	Medical & surgical	Maternity & neonates	In-hospital perinatal mortality	Maternal & neonatal infections, emergency readmissions and injuries
Pasupathy 2010 [73] [3]	UK (Scotland) [NR]	1985 to 2004	1,039,560	Spontaneous & elective	Medical & surgical	Maternity & neonates	Neonatal mortality (within first week of birth)	-
Salihu 2012 [74] [4]	USA (Missouri)	1989 to 1997	Not stated	Spontaneous & elective	Medical & surgical	Maternity & neonates	Neonatal, post-neonatal and infant death	-[d]
Snowden 2013 [75] [4]	USA (California) [257]	2006	462,322	Spontaneous & elective	Medical & surgical	Maternity	-	Birth asphyxia

Study [rating of statistical adjustment][$]	Country [number of hospitals]	Study period	Sample size (admissions unless otherwise stated)	Type of admission (emergency, elective)	Type of procedure (medical, surgical, maternity)	Population	Mortality outcomes	Other outcomes: Adverse events Length of stay Patient satisfaction
Snowden 2016 [42] [4]	USA (California) [214]	2009 to 2010	724,967	Spontaneous & elective	Medical & surgical	Maternity	Neonatal death (timing not specified)	Adverse maternal and neonatal outcomes (including prolonged length of stay)
Wu 2011 [76] [4]	USA (California) [NR]	1999 to 2002	1,864,766	Spontaneous & elective	Medical & surgical	Maternity	-	Neonatal encephalopathy
**Other**								
Buckley 2012 [77] [4]	Australia (New South Wales) [63]	2006 to 2010	4,370 clinical management incidents	Unclear	Unclear	Unclear	-	Adverse events

[$] [1] Comprehensive adjustment; [2a] adequate adjustment – acute physiology; [2b] adequate adjustment – contextual factors; [3] partial adjustment; [4] inadequate adjustment. See Appendix 1, page 11 for further detail.

* Not included in meta-analyses due to lack of required data (e.g. variance/standard errors of the estimates and sample size)

^ For each patient, only the last emergency admission during the study period was included

[a] Rated 2b for stratified analyses that were restricted to admissions through the Emergency Department, as the contextual factor (route of admission) was accounted for in this analysis

[b] only crude (unadjusted) data were reported for overall weekend effect; adjusted analyses were reported only for specific conditions which were not included in meta-analyses of this review

[c] Statistical adjustment was poorly reported in one of the articles [60]

[d] Reported "labour complications" (e.g. placenta abruption and placenta praevia), but these were adverse maternal outcomes associated with maternal conditions and are not considered adverse events as defined in this review.

ED: emergency department; ICU: intensive care unit; NR: not reported; VA: Veterans Affairs

Appendix 7. Technical information for Bayesian meta-analysis

7.1 Trace-plots and pairs plots for primary Bayesian meta-analysis

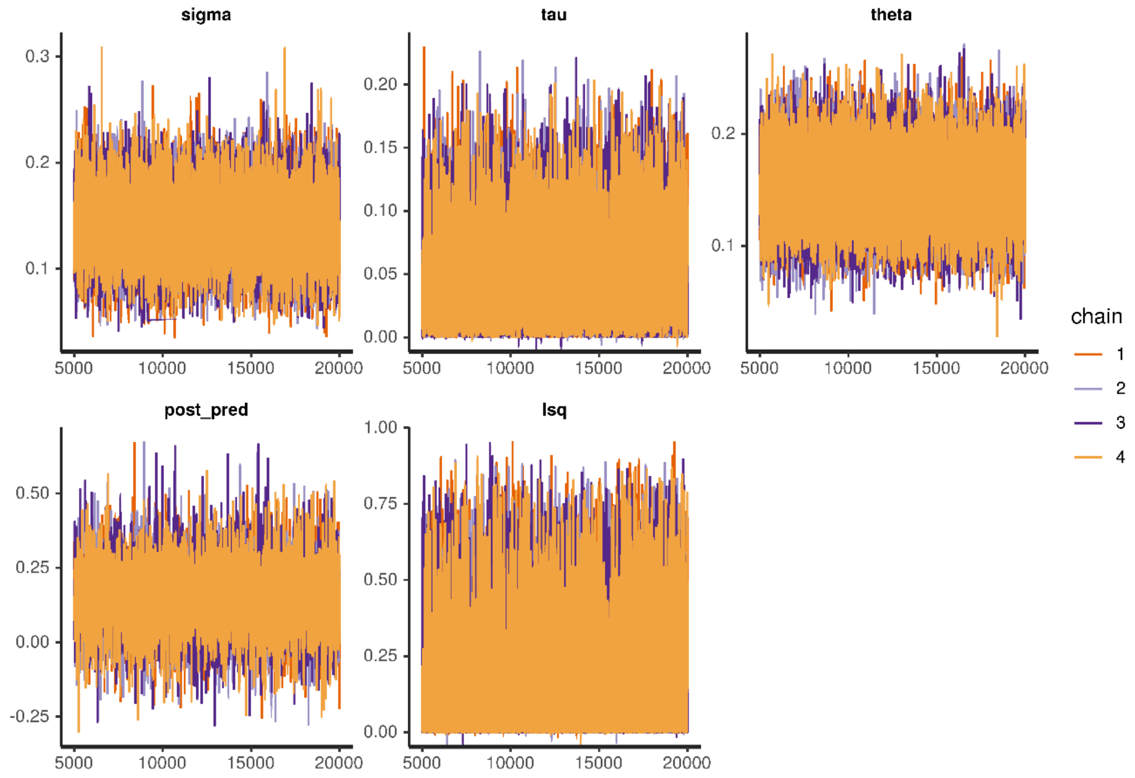

Figure 2 Trace-plots for primary Bayesian meta-analysis

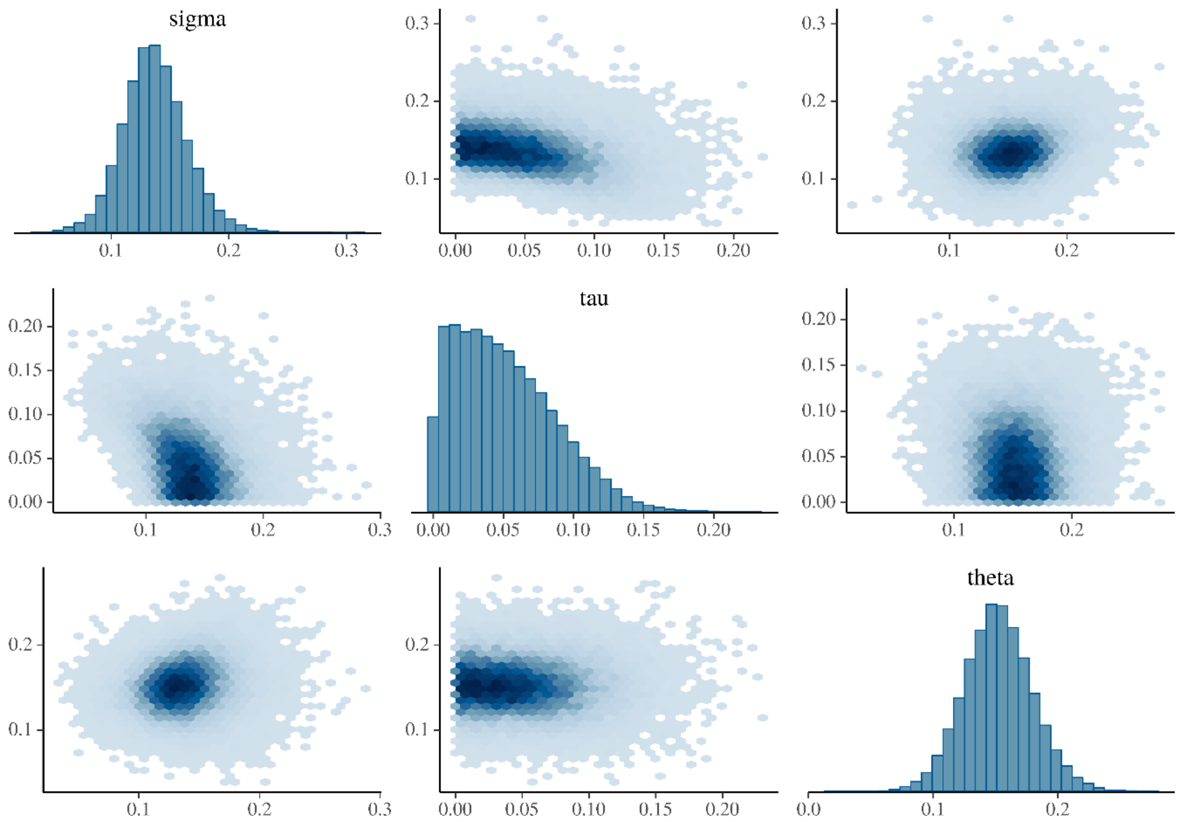

Figure 3 Pairs plots for primary Bayesian meta-analysis

7.2 Statistical outputs for primary Bayesian meta-analysis and sensitivity analysis

Table 6 Statistical outputs for primary Bayesian meta-analysis

	Mean	SD	2.5%	97.5%	N_eff	Rhat
Sigma	0.14	0.03	0.09	0.19	14,558	1.00
Tau	0.05	0.04	0.00	0.13	5,611	1.00
Theta	0.15	0.03	0.10	0.21	25,729	1.00
Posterior predictive	0.15	0.07	0.00	0.29	48,059	1.00
I^2	0.16	0.17	0.00	0.62	5,848	1.00

N: 44, Pooled mean: 1.16 (1.10, 1.23), Posterior predictive mean: 1.16 (1.00, 1.34), I-squared:[1] 16% (0%, 62%). 20,000 iterations, 5,000 warmup iterations, 4 chains. SD: standard deviation. N eff: effective sample size.

Table 7 Statistical outputs for Bayesian sensitivity analysis (allowing partial overlap between studies)

	Mean	SD	2.5%	97.5%	N_eff	Rhat
Sigma	0.17	0.03	0.12	0.22	7,519	1.00
Tau	0.07	0.03	0.01	0.14	2,945	1.00
Theta	0.14	0.03	0.09	0.20	13,566	1.00
Posterior predictive	0.14	0.09	-0.03	0.32	27,611	1.00
I^2	0.18	0.13	0.00	0.48	3,472	1.00

N: 77, Pooled mean: 1.15 (1.10, 1.22), Posterior predictive mean: 1.16 (0.97, 1.39), I-squared: 18% (0%, 48%). 10,000 iterations, 2,000 warmup iterations, 4 chains SD: standard deviation. N eff: effective sample size.

[1] The I-squared statistic is equivalent to the ratio of between-study to total variance in a 2-level model. For our 3-level analysis (within analysis, between-analysis within-study, between study), it is equivalent to the ratio of between-study to total variance. But this statistic is typically biased and shows poor small sample performance, as well as large uncertainty. Any conclusions based on this statistic should be strictly limited.

7.3 Statistical outputs for Bayesian subgroup analyses

Table 8 Statistical outputs for subgroup analysis: All admissions

	Mean	SD	2.5%	97.5%	N_eff	Rhat
Sigma	0.05	0.02	0.03	0.09	9,316	1.00
Tau	0.03	0.03	0.00	0.09	7,023	1.00
Theta	0.12	0.02	0.08	0.16	10,983	1.00
Posterior predictive	0.12	0.04	0.04	0.20	21,241	1.00
I^2	0.19	0.21	0.00	0.74	7,742	1.00

N: 18, Pooled mean: 1.13 (1.09, 1.18), Posterior predictive mean: 1.13 (1.04, 1.22), I-squared: 19% (0%, 74%). 10,000 iterations, 2,000 warmup iterations, 4 chains. S D: standard deviation. N eff: effective sample size.

Table 9 Statistical outputs for subgroup analysis: Emergency admissions

	Mean	SD	2.5%	97.5%	N_eff	Rhat
Sigma	0.07	0.02	0.04	0.12	4,673	1.00
Tau	0.07	0.04	0.00	0.13	3,099	1.00
Theta	0.10	0.02	0.06	0.15	20,580	1.00
Posterior predictive	0.10	0.08	-0.06	0.27	31,343	1.00
I^2	0.44	0.29	0.00	0.90	3,000	1.00

N: 32, Pooled mean: 1.11 (1.06, 1.16), Posterior predictive mean: 1.11 (0.94, 1.31), I-squared: 44% (0%, 90%). 10,000 iterations, 2,000 warmup iterations, 4 chains. S D: standard deviation. N eff: effective sample size.

Table 10 Statistical outputs for subgroup analysis: Elective admissions

	Mean	SD	2.5%	97.5%	N_eff	Rhat
Sigma	0.33	0.13	0.13	0.65	11,180	1.00
Tau	0.32	0.23	0.02	0.92	10,572	1.00
Theta	0.53	0.21	0.07	0.92	13,249	1.00
Posterior predictive	0.53	0.45	-0.45	1.41	24,310	1.00
I^2	0.44	0.29	0.00	0.93	8,769	1.00

N: 12, Pooled mean: 1.70 (1.08, 2.52), Posterior predictive mean: 1.70 (0.64, 4.11), I-squared: 44% (0%, 93%). 10,000 iterations, 2,000 warmup iterations, 4 chains. SD: standard deviation. N eff: effective sample size.

Table 11 Statistical outputs for subgroup analysis: Maternity admissions

	**Mean**	**SD**	**2.5%**	**97.5%**	**N_eff**	**Rhat**
Sigma	0.11	0.11	0.00	0.38	8,923	1.00
Tau	0.11	0.11	0.00	0.38	8,497	1.00
Theta	0.06	0.09	-0.11	0.25	10,674	1.00
Posterior predictive	0.06	0.18	-0.28	0.43	21,339	1.00
I^2	0.44	0.32	0.00	0.96	10,496	1.00

N: 6, Pooled mean: 1.06 (0.89, 1.29), Posterior predictive mean: 1.06 (0.75, 1.53), I-squared: 44% (0%, 96%). 10,000 iterations, 2,000 warmup iterations, 4 chains. SD: standard deviation. N eff: effective sample size.

Appendix 8. Sensitivity analyses

8.1 Sensitivity analyses for the primary meta-analysis

Our primary meta-analysis was conducted using the best adjusted, non-overlapping data from individual studies to avoid double counting. As shown in Appendix 4, data from many studies included in this review were potentially overlapping (i.e. they were based on the same admissions) and these were excluded. As the degree of overlapping between studies varies, the primary analysis may have discarded some useful information. We therefore performed a sensitivity analysis that included these additional data by relaxing our rule and allowing for some overlapping of data between studies. For studies/articles that are based on entirely overlapping or the same dataset, the rule of using the best adjusted effect estimate still applies here. The result of the sensitivity analysis is shown in the table below.

To explore potential small study effects (i.e. studies of smaller sample sizes reporting larger effects), we constructed a funnel plot, which is shown in Figure 4 below. Some level of asymmetry was observed in the plot.

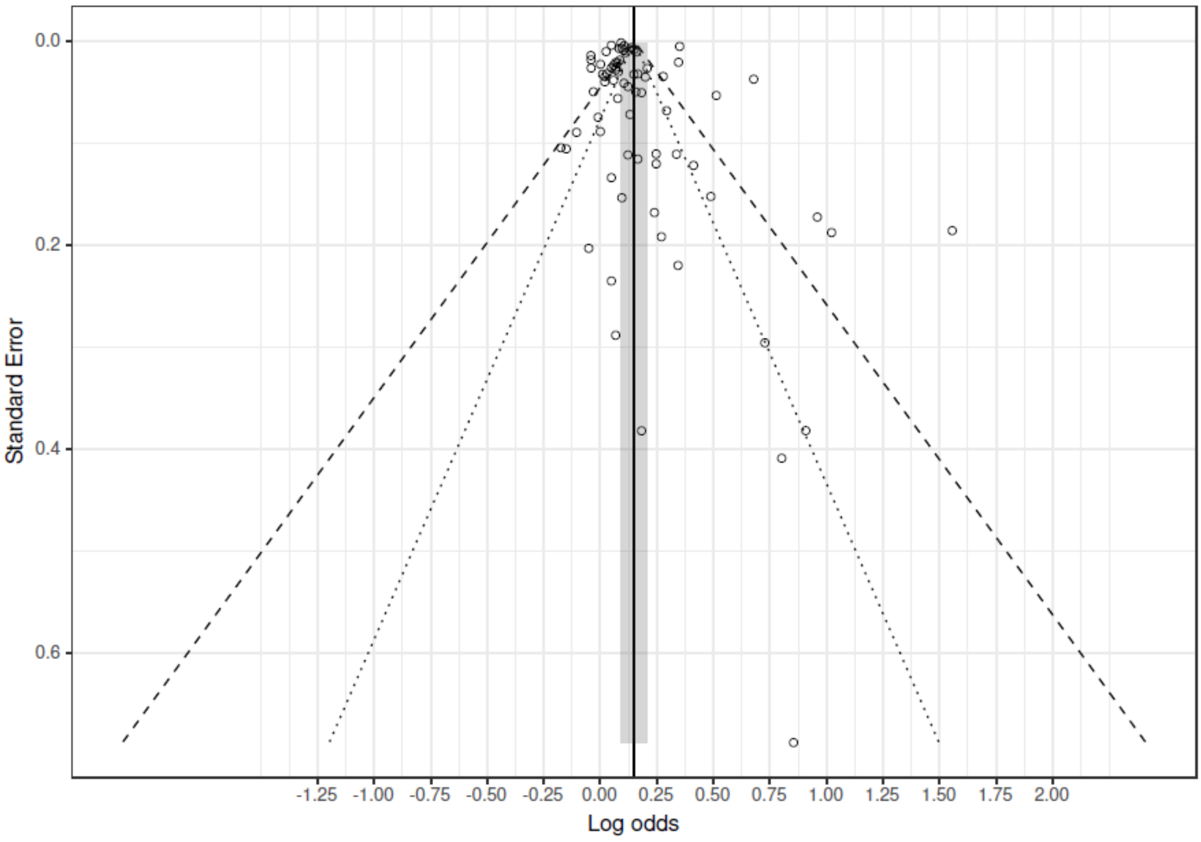

Figure 4 Funnel plot for the weekend effect on mortality for all types of admissions

In view of the apparent asymmetry of the funnel plot, we used data augmentation to explore the potential impact on the estimated weekend effect if the funnel plot asymmetry was caused by publication bias.[78] Data augmentation is a method that can be used to 'adjust for ' potential publication bias by assuming that observation of a study is determined by its p-value alone. P-values are divided into different categories, e.g. [0 to 0.1], [0.1 to 0.5]… and within each category the probability of observing a study (identifying the study and including it in a systematic review) can be different, for example studies that fall into a small p-value category (i.e. studies with a statistically highly significant result) are more likely to be published (and hence be 'observed') than those fall into a larger p-value category (i.e. studies with statistically non-significant results). Findings from repeating our primary meta-analysis and sensitivity analysis using data augmentation are presented in Table 12 below, and statistical outputs from these analyses are provided in Table 13 and Table 14.

The analyses adopted the following three categories: [0 to 0.01], [0.01 to 0.05], [0.05 to 1]. The results show that augmentation slightly reduced the estimated weekend effect in each cases.

Table 12 Results of data augmentation analyses for the primary and sensitivity meta-analysis

**Sensitivity analyses**	**N**	**Pooled mean (95% CrI)**	**Posterior predictive mean (95% CrI)**	**I^2 (95% CrI)**
Primary meta-analysis (all types of admissions)	44	1.16 (1.10, 1.23)	1.16 (1.00, 1.34)	0.16 (0.00, 0.62)
Primary meta-analysis with data augmentation, using 3 p-value categories		1.11 (1.08, 1.13)	-	-
Sensitivity analysis (all types of admissions, allowing overlap of data between studies)	77	1.15 (1.10, 1.22)	1.16 (0.97, 1.39)	0.18 (0.00, 0.48)
Sensitivity analysis with data augmentation, 3 p-value categories		1.12 (1.09, 1.14)	-	-

N: number of observations (estimates of the weekend effect from individual studies). CrI: credible interval.

Table 13 Bayesian statistical outputs for primary meta-analysis with data augmentation

	**Mean**	**SD**	**2.5%**	**97.5%**	**N_eff**	**Rhat**
Tau	0.10	0.01	0.08	0.12	40,984	1.00
Theta	0.10	0.01	0.09	0.12	37,370	1.00

Estimated pooled effect 1.11 (1.08, 1.13). Three p-value categories used: [0 to 0.01], [0.01 to 0.05], [0.05 to 1]. SD: standard deviation. N eff: effective sample size.

Table 14 Bayesian statistical outputs for sensitivity meta-analysis (allowing partial overlap of data between studies) with data augmentation

	**Mean**	**SD**	**2.5%**	**97.5%**	**N_eff**	**Rhat**
Tau	0.11	0.01	0.09	0.13	48,825	1.00
Theta	0.12	0.01	0.11	0.14	50,184	1.00

Estimated pooled effect 1.12 (1.09, 1.14). Three p-value categories used: [0 to 0.01], [0.01 to 0.05], [0.05 to 1]. SD: standard deviation. N eff: effective sample size.

Appendix 9. Subgroup analyses (mortality)

9.1 Subgroup analyses by types of admissions

Forest plots for the pre-specified subgroup analyses based on types of admissions included in each study are shown below.

9.1.1 All admissions

Study	Country	Study period	Adequacy of adjustment	Estimated OR
Ruiz 2015	Australia	2009 to 2012	3	1.03 (0.96, 1.10)
Ruiz 2015	Australia	2009 to 2012	3	1.03 (0.97, 1.10)
Madsen 2014	Denmark	1995 to 2012	4	2.23 (1.00, 4.97)
Lee 2012	Malaysia	2008 to 2010	4	1.22 (1.14, 1.31)
Ruiz 2015	Netherland	2009 to 2012	3	1.25 (1.13, 1.37)
Ruiz 2015	Netherland	2009 to 2012	3	1.32 (1.20, 1.45)
Freemantle 2012	UK	2009 to 2010	2b	1.16 (1.14, 1.18)
Freemantle 2012	UK	2009 to 2010	2b	1.11 (1.09, 1.13)
Freemantle 2015	UK	2013 to 2014	2b	1.10 (1.08, 1.11)
Freemantle 2015	UK	2013 to 2014	2b	1.15 (1.14, 1.17)
Mohammed 2012	UK	2008 to 2009	3	1.14 (1.10, 1.18)
Attenello 2015	USA	2002 to 2010	2b	1.11 (1.10, 1.12)
Conway 2017b	USA	1998	3	1.12 (1.09, 1.14)
Freemantle 2012	USA	2010	2b	1.18 (1.11, 1.26)
Freemantle 2012	USA	2010	2b	1.16 (1.09, 1.24)
Gould 2003	USA	1995 to 1997	3	1.01 (0.95, 1.08)
Ruiz 2015	USA	2009 to 2012	3	1.13 (1.04, 1.24)
Ruiz 2015	USA	2009 to 2012	3	1.12 (1.03, 1.21)
**Pooled**				**1.13 (1.09, 1.18)**
**Posterior predictive**				**1.13 (1.04, 1.22)**

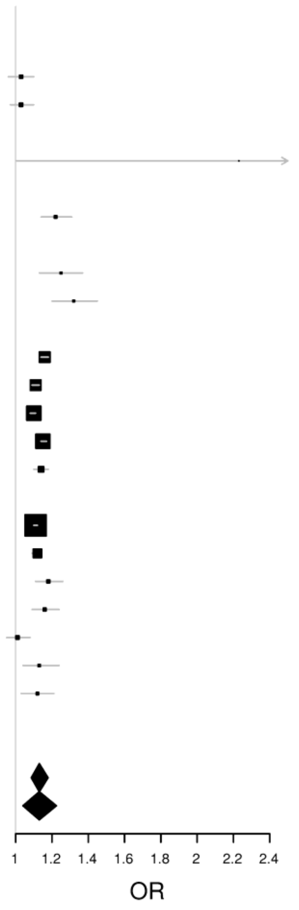

Note: some of the studies reported two separate estimates of the weekend effect for a given country, for example Saturday vs. weekday(s) and Sunday vs. weekday(s). Both estimates were included in the meta-analysis as they provided additional information while the correlation between the estimates within individual studies was accounted for in the multi-level Bayesian model. The study by Ruiz et al. 2015 provided estimates for multiple countries.

Figure 5 Weekend effect on mortality in studies covering all admissions (including both medical and surgical, emergency and elective admissions)

9.1.2 Emergency admissions

Study	Country	Study period	Adequacy of adjustment	Estimated OR
Perez Concha 2014	Australia	2000 to 2007	3	1.17 (1.14, 1.19)
Ruiz 2015	Australia	2009 to 2012	3	1.03 (0.97, 1.10)
Ruiz 2015	Australia	2009 to 2012	3	1.02 (0.95, 1.09)
Conway 2016	Ireland	2002 to 2014	2a	1.02 (0.94, 1.10)
De Giorgi 2015	Italy	2000 to 2013	4	1.41 (1.36, 1.47)
Ruiz 2015	Netherland	2009 to 2012	3	1.20 (1.09, 1.33)
Ruiz 2015	Netherland	2009 to 2012	3	1.17 (1.06, 1.29)
Barba 2006	Spain	1999 to 2003	3	1.08 (0.97, 1.21)
Lee 2006	Taiwan	2000 to 2002	3	0.96 (0.93, 0.99)
Aylin 2010	UK	2005 to 2006	3	1.10 (1.08, 1.11)
Freemantle 2012	UK	2009 to 2010	2b	1.14 (1.12, 1.16)
Freemantle 2012	UK	2009 to 2010	2b	1.11 (1.09, 1.13)
Gillies 2017	UK	2005 to 2007	3	1.14 (0.98, 1.30)
Han 2017a	UK	2004 to 2014	2b	1.08 (1.02, 1.15)
Handel 2012	UK	1999 to 2009	4	1.42 (1.40, 1.43)
Maggs 2010	UK	2007 to 2008	4	1.11 (0.97, 1.25)
McCallum 2016	UK	2000-2014	2b	1.00 (0.84, 1.19)
McCallum 2016	UK	2000 to 2014	2b	0.90 (0.76, 1.08)
Meacock 2016	UK	2013 to 2014	2b	1.05 (1.04, 1.07)
Meacock 2016	UK	2013 to 2014	2b	1.21 (1.16, 1.26)
Mohammed 2012	UK	2008 to 2009	3	1.09 (1.05, 1.13)
Mohammed 2017	UK	2014	2a	0.97 (0.88, 1.07)
Roberts 2015	UK	2004 to 2012	4	1.09 (1.07, 1.10)
Shiue 2017	UK	2010 to 2015	4	1.06 (1.01, 1.11)
Walker 2017	UK	2006 to 2014	1	1.07 (1.01, 1.13)
Walker 2017	UK	2006 to 2014	1	1.05 (1.00, 1.11)
An 2017	USA	2003 to 2013	3	1.05 (1.04, 1.05)
Cram 2004	USA	1998	2b	1.03 (1.01, 1.06)
Goldstein 2014	USA	1988 to 2010	2b	1.63 (1.21, 2.20)
Ruiz 2015	USA	2009 to 2012	3	1.11 (1.02, 1.20)
Ruiz 2015	USA	2009 to 2012	3	1.13 (1.04, 1.24)
Zapf 2015	USA	2007 to 2010	3	1.27 (0.91, 1.76)
**Pooled**				**1.11 (1.06, 1.16)**
**Posterior predictive**				**1.11 (0.94, 1.31)**

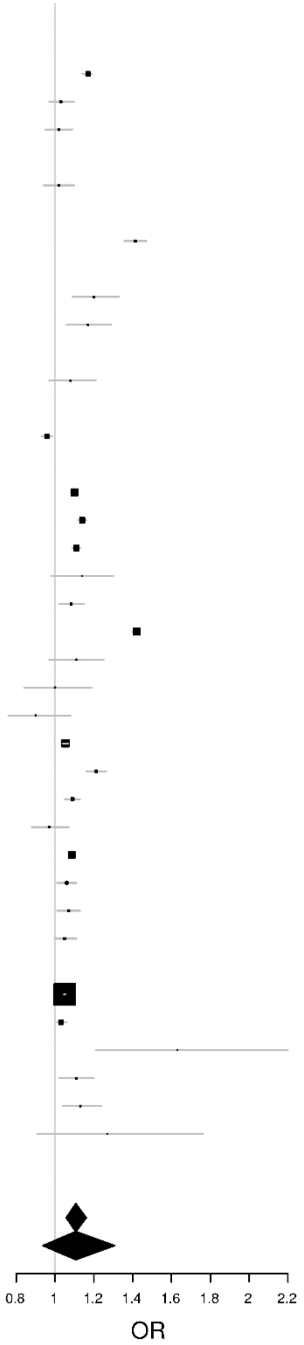

Note: some of the studies reported two separate estimates of the weekend effect for a given country, for example Saturday vs. weekday(s) and Sunday vs. weekday(s). Both estimates were included in the meta-analysis as they provided additional information while the correlation between the estimates within individual studies was accounted for in the multi-level Bayesian model. The study by Ruiz et al. 2015 provided estimates for multiple countries

Figure 6 Weekend effect on mortality in studies covering emergency admissions

9.1.3 Elective admissions

Study	Country	Study period	Adequacy of adjustment	Estimated OR
Ruiz 2015	Australia	2009 to 2012	3	1.20 (0.57, 2.55)
Ruiz 2015	Australia	2009 to 2012	3	2.07 (1.16, 3.70)
McIsaac 2014	Canada	2002 to 2012	2b	1.51 (1.19, 1.92)
Ruiz 2015	Netherland	2009 to 2012	3	4.74 (3.29, 6.82)
Ruiz 2015	Netherland	2009 to 2012	3	2.61 (1.86, 3.66)
Freemantle 2012	UK	2009 to 2010	2b	1.62 (1.50, 1.75)
Freemantle 2012	UK	2009 to 2010	2b	1.18 (1.09, 1.28)
Mohammed 2012	UK	2008 to 2009	3	1.32 (1.23, 1.41)
Ruiz 2016	UK	2008 to 2011	2b	1.97 (1.83, 2.12)
Ruiz 2016	UK	2008 to 2011	2b	1.67 (1.50, 1.85)
Ruiz 2015	USA	2009 to 2012	3	2.48 (1.17, 5.23)
Ruiz 2015	USA	2009 to 2012	3	2.35 (0.61, 9.04)
**Pooled**				**1.70 (1.08, 2.52)**
**Posterior predictive**				**1.70 (0.64, 4.11)**

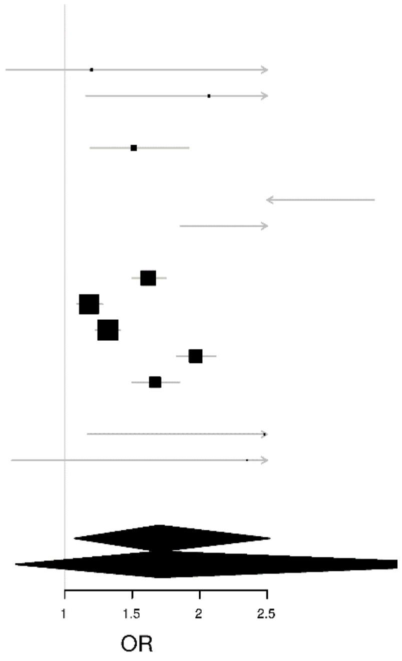

Note: some of the studies reported two separate estimates of the weekend effect for a given country, for example Saturday vs. weekday(s) and Sunday vs. weekday(s). Both estimates were included in the meta-analysis as they provided additional information while the correlation between the estimates within individual studies was accounted for in the multi-level Bayesian model. The study by Ruiz et al. 2015 provided estimates for multiple countries

Figure 7 Weekend effect on mortality in studies covering elective surgical admissions

9.1.4 Maternity admissions

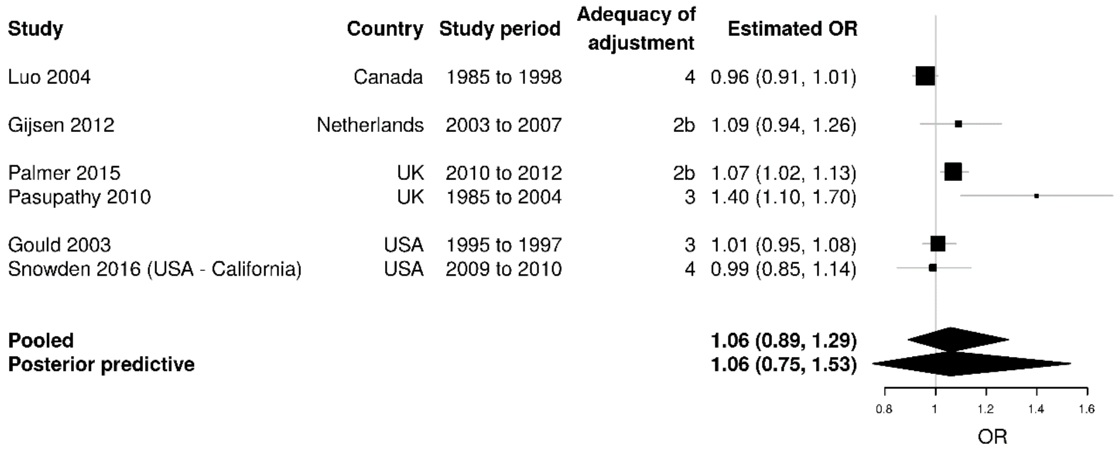

Figure 8 Weekend effect on mortality in studies covering maternity admissions

9.1.5 Within study comparisons: emergency vs elective admissions

Table 15 Estimated weekend effects for emergency and elective admissions in studies where both were reported

Study & location	Emergency admissions	Elective admissions
Mohammed et al. 2012 [15]<br>England (nationwide)	1.09 (1.05 to 1.13)	1.32 (1.23 to 1.41)
Ruiz et al. 2015 [19]		
Australia (6 hospitals)	1.02 (0.95 to 1.09)	2.07 (1.16 to 3.70)
England (11 hospitals)	1.08 (1.04 to 1.13)	2.78 (1.93 to 4.03)
Netherlands (6 hospitals)	1.17 (1.06 to 1.29)	2.61 (1.86 to 3.66)
USA (5 hospitals)	1.13 (1.04 to 1.24)	2.35 (0.61 to 9.04)

Data shown are adjusted odds ratios and their 95% confidence intervals reported in the individual studies

9.1.6 Emergency admissions through Accident & Emergency (A&E) department

Table 16 Estimated weekend effects for different subgroups of emergency admissions based on route of admission

	Ratio of weekend to weekday admissions	Mortality (%) weekend	Mortality (%) weekday	Unadjusted OR	Adjusted OR
Cram et al. 2004,[32] in-hospital mortality					
All admissions	0.26	6.7	5.7	1.20 (1.17 to 1.23)	1.12 (1.09 to 1.14)
Unscheduled admissions only	0.31	6.7	6.0	1.14 (1.11 to 1.16)	1.10 (1.07 to 1.12)
A&E admissions only	0.39	6.7	6.4	1.03 (1.01 to 1.06)	1.03 (1.01 to 1.06)
Meacock et al. 2016,[26] 30-day mortality					
Direct admission from community	N/A	2.72	2.37	N/A	1.21 (1.16 to 1.26)
Admissions through A&E	N/A	3.59	3.42	N/A	1.05 (1.04 to 1.07)
Sharp et al. 2013,[41] in-hospital mortality					
Mortality in the A&E or following admissions through A&E	N/A	4.23	3.96	1.07 (1.06 to 1.08)	1.03 (1.01 to 1.05)
Mortality following admission through A&E	N/A	N/A	N/A	N/A	1.03 (1.01 to 1.05)

9.2 Mortality – subgroups by time period

This is partly dealt with in meta-regression but this could be confounded by study-level variables, Below is a summary of within-study observations as triangulation of this finding from meta-regression.

Table 17 Studies in which changes in the weekend effect over time were explored and their findings

Study, location, statistical adjustment	Type of admissions & outcome measure	Changes in the weekend effect over time
Database studies		
An 2017 [39] (USA – Nationwide) [3]	Emergency admissions (both medical & surgical); in-hospital mortality	Significant reduction between 2003 (HR 1.069, 1.053 to 1.084) and 2013 (HR 1.025, 1.010 to 1.040)
Handel et al. 2012 [54] (UK – Scotland) [4]	Emergency admissions (both medical & surgical); in-hospital mortality	Examined the weekend effect for each year between 1999 and 2009, and found it stayed 'much the same' during this period (OR fluctuated between 1.35 and 1.46)
Luo et al. 2004 [71] (Canada – nationwide) [4]	Maternity admissions; stillbirth and early neonatal mortality (0-6 days)	Stated "the slightly elevated crude risks of overall stillbirth and overall early neonatal death for infants born on weekends persisted through 1985–1989, 1990–1994 and 1995–1998".
McCallum et al. 2016 [8] (UK - England) [2b]	Emergency general surgical admissions; 30-day, in-hospital mortality	Reported that the weekend effect "was consistent" across three time intervals when calculated according to the date of admission, but it was reduced in the period 2010-2014 compared with the period 2000-2004 when calculated according to the date of operation.
McIsaac et al. 2014 [67] (Canada - Ontario) [2b]	Elective, non-cardiac surgical admissions; 30-day mortality (both in/out of hospital)	Reported similar estimates of the weekend effect for 2002-2007 (OR 1.78, 1.13 to 2.84) and 2008-2012 (OR 1.60, 0.89 to 2.85).
Meacock & Sutton 2017 [27] (UK – England) [3]	Emergency admissions; 30-day mortality	Reported an average change of 0.004 (in the odds ratio, 95% CI −0.017 to 0.025) over time between 2015/2016 and 2013/2014 in the estimated weekend effect among 123 trusts (hospital/hospital groups). Substantial variations in these changes were observed (SD=0.118) among individual trusts, with the change ranging from a decrease of 0.340 to an increase of 0.380.

Roberts et al. 2015 [9] (UK – England & Wales) [4]	Emergency admissions; 30-day mortality	Reported similar estimates of the weekend effect over 4 periods (2004-6, 2007-8, 2009-10, 2011-12) for England (OR fluctuated between 1.096 and 1.108) and Wales (OR fluctuated between 1.064 to 1.106)
Single hospital studies		
Conway et al. 2017b [60] (Ireland, single hospital) [4]	Emergency medical admissions; 30-day in-hospital mortality	Analysed three time periods (2002-5, 2006-9, 2010-14) and reported significantly lower weekend effect in more recent period (time period effect OR 0.71, 0.67 to 0.74) and stated that the weekend effect diminishes from OR 1.15 (1.05 to 1.24) to OR 0.90 (0.71 to 1.12) when the interaction between time period and weekend/weekday admission was accounted for.
Lee et al. 2012 [47] (Malaysia, single hospital) [4]	All admissions; in-hospital mortality	Weekend effect reduced over a 3-year period from OR 1.30 (1.15 to 1.47) in 2008 to 1.14 (1.02 to 1.29) in 2010.
Sullivan et al. 2016 [57] (Australia, single hospital) [4]	Emergency admissions; in-hospital mortality	Unadjusted RR 1.28 in 2011 and 1.18 in 2013.

CI: confidence interval; HR: hazard ratio; OR: odds ratio; RR: risk ratio; SD: standard deviation.

9.3 Mortality – subgroups by country

As illustrated in Figure 1 the main text and other forest plots in Appendix 9.1 above, the weekend effect appears to vary between studies undertaken in different countries. Two studies provided data of cohorts from different countries. In a Global Comparators Project, Ruiz et al. investigated 30-day in-hospital mortality for emergency admissions and elective surgical admissions using data from four countries (Table 15 in Appendix 9.1.5 above).[19] Weekend effect was found across the countries and type of admissions, but there were notable variations between the countries and no apparent weekend effect was observed in Australia for emergency admissions in their primary analysis. By contrast, Freemantle and colleagues obtained very similar estimates for two independent datasets from England and USA (Figure 1 in the main text).[18]

9.4 Mortality – subgroups by disease conditions

Although systematic reviews of the weekend effect for individual disease conditions have been published,[79-82] comparisons of the weekend effect between different disease conditions could be confounded by differences in study-level characteristics between studies. Several studies included in this review reported weekend effects by selected, individual disease conditions and they provide a chance to make such a comparison that is less susceptible to confounding by study-level variables. The data are presented in this section. We selected conditions for which the mortality is likely to be affected by hospital staffing level (ruptured abdominal aortic aneurysm, acute epiglottitis, and pulmonary embolism) and those for which mortality is unlikely to be influenced by staffing level as originally hypothesised by Bell and colleagues in their seminal paper, [52] as well as other conditions that commonly contribute to death during hospital admissions.

Overall the estimated weekend effect from different studies are fairly consistent for most of the conditions, but discrepancies exist and the findings do not necessarily agree with hypotheses initially set out by Bell and colleague. A finding worth highlighting is that in the only study (Walker et al) that was judged to have achieved comprehensive statistical adjustment,[13] the test for interaction showed no significant difference (p=0.86) in the estimated weekend effect between admissions associated with different conditions based on the Clinical Classification Software (CCS) groups.

Table 18 Estimated weekend effects on mortality for admissions associated with specific conditions

Condition	Bell et al. 2001 [52] Emergency admissions (odds ratio), weekend vs weekday, Ontario	Aylin et al. 2010 [11] Emergency admissions (odds ratio), weekend vs weekday, England	Cram et al. 2004 [32] - All admissions - Unscheduled admissions; - Unscheduled admissions through A&E (odds ratio), weekend vs weekday, California	Freemantle et al. 2012 [18] All admissions (hazard ratio) Sunday vs Wednesday, England	Roberts et al. 2015 [9] Emergency admissions (odds ratio), weekend vs weekday, England	Roberts et al. 2015 [9] Emergency admissions (odds ratio), weekend vs weekday, Wales
Ruptured abdominal aortic aneurysm*	1.28 (1.13 to 1.46)	Aortic, peripheral and visceral artery aneurysms 1.45 (1.26 to 1.66)	Aortic aneurysm<br>2.13 (1.77 to 2.58)<br>1.38 (1.13 to 1.69)<br>1.13 (0.90 to 1.41)	NR	Abdominal aortic aneurysm 1.510 (1.424 to 1.601)	Abdominal aortic aneurysm 1.945 (1.548 to 2.440)
Acute epiglottitis*	5.28 (1.01 to 27.50)	NR	NR	NR	NR	NR
Pulmonary embolism*	1.19 (1.03 to 1.36)	NR	1.42 (1.15 to 1.76)<br>1.36 (1.09 to 1.70)<br>1.22 (0.59 to 1.60)	NR	1.197 (1.144 to 1.252)	1.245 (1.021 to 1.518)
Acute myocardial infarction^	1.03 (1.00 to 1.06)	1.08 (1.03 to 1.14)	1.09 (1.00 to 1.17)<br>1.04 (0.97 to 1.12)<br>1.01 (0.93 to 1.10)	1.11 (1.01 to 1.23)	1.059 (1.037 to 1.082)	1.040 (0.960 to 1.126)
Intracerebral haemorrhage^	1.01 (0.93 to 1.11)	Acute cerebrovascular disease 1.13 (1.09 to 1.18)	1.20 (1.08 to 1.34)<br>1.11 (1.00 to 1.23)<br>0.98 (0.87 to 1.10)	Acute cerebrovascular disease 1.16 (1.09 to 1.23)	Stroke 1.115 (1.099 to 1.132)	Stroke 1.193 (1.125 to 1.265)
Acute hip fracture^	0.97 (0.90 to 1.04)	Fracture of neck of femur (hip) 0.98 (0.92 to 1.04)	Hip fracture<br>1.13 (0.97 to 1.32)<br>1.14 (0.97 to 1.34)<br>1.13 (0.93 to 1.36)	Fracture of neck of femur (hip) 1.07 (0.95 to 1.19)	Hip fracture 1.019 (0.994 to 1.044)	Hip fracture 1.086 (0.983 to 1.200)
Chronic airway obstruction	1.01 (0.94 to 1.09)	Chronic obstructive pulmonary disease and bronchiectasis 1.00 (0.94 to 1.05)	0.88 (0.63 to 1.24)<br>1.02 (0.67 to 1.56)<br>1.07 (0.61 to 1.86)	Chronic obstructive pulmonary disease 1.02 (0.93 to 1.13)	Chronic obstructive pulmonary disease 1.035 (1.015 to 1.056)	Chronic obstructive pulmonary disease 1.067 (0.990 to 1.150)

Cancer of the trachea, bronchus, or lung	1.19 (1.12 to 1.25)	Cancer of bronchus, lung 1.34 (1.24 to 1.44)	1.51 (1.31 to 1.73)<br>1.20 (1.04 to 1.39)<br>1.11 (0.93 to 1.32)	Cancer of bronchus, lung<br>1.28 (1.16 to 1.43)	NR	NR
Heart failure	1.00 (0.96 to 1.04)	Congestive heart failure non-hypertensive<br>1.11 (1.05 to 1.17)	1.03 (0.95 to 1.12)<br>1.06 (0.98 to 1.16)<br>1.05 (0.96 to 1.16)	Congestive heart failure<br>1.10 (1.01 to 1.21)	1.134<br>(1.112 to 1.156)	1.092<br>(1.011 to 1.178)
Gastrointestinal haemorrhage	1.08 (0.96 to 1.20)	1.08 (1.00 to 1.17)	1.28 (1.04 to 1.57)<br>1.27 (1.04 to 1.57)<br>1.14 (0.92 to 1.42)	NR	Upper GI bleeding<br>1.124<br>(1.094 to 1.155)	Upper GI bleeding<br>1.138<br>(1.017 to 1.274)
Cardiac dysrhythmia	1.17 (1.09 to 1.25)	1.31 (1.17 to 1.47)	1.28 (1.08 to 1.50)<br>1.24 (1.09 to 1.42)<br>1.10 (0.95 to 1.28)	NR	NR	NR
Pneumonia	NR	1.00 (0.97 to 1.04)	0.99 (0.92 to 1.06)<br>1.00 (0.94 to 1.08)<br>0.94 (0.87 to 1.02)	1.03 (0.98 to 1.08)	1.037<br>(1.025 to 1.049)	1.092<br>(1.043 to 1.145)
Septicaemia	0.99 (0.91 to 1.07)	Except in labour<br>1.04 (0.96 to 1.13)	1.10 (1.03 to 1.17)<br>1.09 (1.02 to 1.17)<br>1.04 (0.96 to 1.12)	Except in labour<br>1.07 (0.96 to 1.18)	NR	NR

*Conditions hypothesised by Bell et al. for which a weekend effect is likely to be observed because these conditions: (1) occur frequently; (2) the in-hospital mortality rate among patients with the condition is high; (3) the first few days of hospitalisation are critical; (4) the condition is treatable; (5) care involves logistic difficulties; (6) death can be rapid; (7) patients with the condition typically receive a substantial amount of care in clinical settings other than a critical care unit or A&E.

^Conditions hypothesised by Bell et al. for which a weekend effect is less likely to be observed: The first was acute myocardial infarction, which is usually managed in a critical care setting, where fluctuations in staffing levels are minimal. The second was acute intracerebral haemorrhage, for which effective treatment is generally unavailable. The third was acute hip fracture, a condition that is sometimes treated more promptly on weekends than on weekdays, because operating rooms are more available on weekends.

A & E: accident & emergency; NR: not reported.

9.5 Mortality – correlation of hospital weekend staffing level and the weekend effect

Meacock and Sutton, based on the “experimental statistics” published by the English NHS Digital, reported an average trust (hospital/hospital group) weekend effect of 1.119 (odds ratio, 95% CI not reported, 30-day mortality, 2015-16), with the odds ratio for individual trust ranging from 0.920 to 1.360 (SD=0.081). [27] They examined the correlation between (1) the estimated weekend effect for year 2015-16; (2) the change in the estimated weekend effect from year 2013-14 to 2015-16 for individual trust and four measures of comprehensiveness of acute care in hospital at weekends. These measures are used as clinical standards by the English NHS to monitor the progress of its implementation of 7-day services, and include: (1) time to first consultant review within 14 hours of arrival at hospital; (2) being able to access diagnostic services within 1 hour for critical patients, within 12 hours for urgent patients, and within 24 hours for non-urgent patients 7 days a week; (3) having 24/7 access to consultant directed interventions including critical care, interventional radiology and endoscopy, and emergency general surgery; (4) all patients in critical and acute areas are reviewed twice daily, and those in general wards are reviewed once daily (unless otherwise considered unnecessary), 7 days a week. Neither the estimated weekend effect, nor the change in the estimated weekend effect over time, showed a significant correlation with these four measures. The findings were consistent for both all admissions and emergency admissions

Aldridge et al. carried out a survey of all hospital trusts (groups of hospitals) receiving unselected emergency admissions in England to measure specialist (consultant) intensity on a weekend day (Sunday) and a weekday (Wednesday) in 2014. [24] Specialist intensity was defined as the self-reported estimated number of specialist hours per ten emergency admissions between 08:00 hour and 20:00 hour in each trust. Trust-specific weekend effect on mortality was calculated using the Hospital Episode Statistics, adjusting for age, sex, deprivation, diagnostic category and comorbidity.

Of the 141 eligible trust, 115 (91%) participated with 15537/34350 (45%) of surveyed clinicians responded. The results show that the median specialist intensity reported on Sunday was only 48% of than on Wednesday, but no significant association was found (r = 0.042; p=0.654) between the trust-level Sunday to Wednesday intensity ratio and the weekend effect (which is the adjusted Sunday to Wednesday mortality ratio).

Appendix 10. Impact of variations in methodological approaches

This section examines the potential impact of different methodological approaches on the estimated weekend effect, using data from within-study comparisons (which avoid confounding by study-level variables) where possible.

10.1 Impact of statistical adjustment for acute physiology

Although studies from various research teams have reported that inclusion of measures of acute physiology diminishes (although not necessarily abolish completely) the weekend effect, the impact is not consistent over time and in particular appears to be very sensitive depending on completeness of the data. For example in Walker et al, [13] adjusting for biochemistry and haematological test results slightly increases the estimate weekend effect compared with the model without adjusting for these variables when patients from all emergency admissions were included. The estimated weekend effect substantially reduced among patients who had a complete set of these test results.

Table 19 Reported estimates of the weekend effect before and after adjusting for measures of acute physiology

Publication & location	Study period	Effect measure	Estimated weekend effect (OR) without adjusting for acute physiology	Estimated weekend effect adjusted for acute physiology
Conway et al 2016 [58] (Dublin, Ireland)	2002 to 2014	Odds ratio	1.08 (1.01 to 1.15)	1.07 (0.98 to 1.16)
Conway et al 2017a [59] (Dublin, Ireland)	2002 to 2014	Odds ratio	1.05 (0.97 to 1.13)	1.03 (0.96 to 1.09)
Mikulich et al 2011 [62] (Dublin, Ireland)	2002 to 2009	Odds ratio	1.11 (0.99 to 1.23)	1.05 (0.88 to 1.24)
Mohammed et al 2017 [28] (Yorkshire & Humberside, UK)	2014	Odds ratio	1.10 (1.01 to 1.20)	0.97 (0.88 to 1.07)
Walker et al 2017 [13] (Oxford, UK)	2006 to 2014 All patients	Adjusted relative risk	1.09 (1.03–1.14)	1.11 (1.05 to 1.18)
Walker et al. 2017 [13] (Oxford, UK)	2006 to 2014 Patients with complete laboratory test results	Adjusted relative risk	NR	1.05 (1.00 to 1.11)

NR: not reported

10.2 Different definitions of weekends

Table 20 Studies in which the weekend effect was estimated using different definitions of the weekend

Study & outcome measure	Different definitions of weekends	Estimated weekend effect
Lee et al. 2006* [55]<br>Death within 24 hours	Weekend vs working days<br>Consecutive holiday vs working days	OR 1.005 (0.953 to 1.059)<br>OR 1.150 (1.005 to 1.315)
Lee et al. 2006* [55]<br>Death within 48 hours	Weekend vs working days<br>Consecutive holiday vs working days	OR 1.001 (0.957 to 1.047)<br>OR 1.163 (1.037 to 1.303)
Lee et al. 2006* [55]<br>30-day mortality	Weekend vs working days<br>Consecutive holiday vs working days	OR 0.959 (0.932 to 0.986)<br>OR 1.130 (1.051 to 1.214)
Walker et al. 2017 [13]<br>Saturday vs Wednesday<br>30-day mortality	Days start at midnight<br>Days start at 7 am rather than midnight Days start at 8 am rather than midnight	aRR 1.08 (1.03 to 1.14)<br>aRR 1.11 (1.06 to 1.17)<br>aRR 1.11 (1.06 to 1.17)
Walker et al. 2017 [13]<br>Sunday vs Wednesday<br>30-day mortality	Days start at midnight<br>Days start at 7 am rather than midnight<br>Days start at 8 am rather than midnight	aRR 1.09 (1.03 to 1.14)<br>aRR 1.07 (1.02 to 1.13)<br>aRR 1.08 (1.03 to 1.13)

aRR: adjusted relative risk; OR: odds ratio
*Weekend included Saturday and Sunday. Consecutive holiday included weekend connected with another public holiday

10.3 Different measures for mortality

In general, weekend effect is more profound for short-term mortality than longer-term mortality but there are exceptions. Bell et al. 2001 [52] stated that "analyses of deaths within two days after admission, rather than total in-hospital deaths, generally showed larger relative differences in mortality between weekend and weekday admissions." Perez Concha et al. 2014 showed very similar pattern between in-hospital deaths & post-discharge deaths at 7 days. [56] Walker et al. 2017 found that "in unadjusted models, excess risks associated with weekend admission were greater at shorter timescales; however, after adjusting for administrative factors excess risks associated with emergency admission on Saturdays or Sundays vs Wednesdays were similar for 7-day to 30-day mortality (Supplementary Figure 9(a)). Similarly, adjusting for test results attenuated these excess risks, regardless of timescale over which the mortality outcome was assessed (Supplementary Figure 9(a))." [13]

Table 21 Studies in which the weekend effect was estimated using different mortality measures

Study	Type of admissions	Outcome measure	Mortality measure	Effect estimates
Freemantle et al. 2012 [18]	All admissions	HR<br>HR<br><br>HR	In-hospital mortality<br>In-hospital mortality (death within 3 days of admission censored)<br>30-day mortality (in/out hospital)	1.16 (1.14 to 1.18)<br>1.11 (1.09 to 1.13)<br><br>1.14 (1.13 to 1.16)
Madsen et al. 2014 [48]	All medical admissions	RR<br>RR	In-hospital mortality<br>30-day mortality (in/out hospital)	2.23 (CI not reported)<br>1.77 (CI not reported)
Han et al. 2017 [10]	Emergency medical & surgical	OR<br>OR	In-hospital mortality<br>7-day mortality (in/out hospital)<br>30-day mortality (in/out hospital)	1.083 (1.021 to 1.149)<br>1.122 (1.069 to 1.179)<br>1.104 (1.057 to 1.154)
Barba et al. 2006 [51]	Emergency medical & surgical	OR<br>OR	2-day mortality (in-hospital)<br>"Global mortality"	1.40 (1.20 to 1.61)<br>1.08 (0.97 to 1.21)
Lee et al. 2006 [55]	Emergency medical & surgical	OR<br>OR<br>OR	1-day mortality (in/out hospital)<br>2-day mortality (in/out hospital)<br>30-day mortality (in/out hospital)	1.005 (0.953 to 1.059)<br>1.001 (0.957 to 1.047)<br>0.959 (0.932 to 0.986)
Walker et al. 2017 [13]	Emergency medical & surgical	aRR	7-day, 14-day, 21-day and 30-day mortality (in/out hospital)	Data presented in graphs; overall very similar between the different measures
Gillies et al. 2017 [65]	Emergency general surgical	OR<br>HR	Perioperative mortality (30-day)<br>Overall survival (death from any cause)	1.14 (0.98 to 1.30)<br>1.00 (0.96 to 1.04)
Ozdemir et al. 2016 [12]	Emergency (general) surgical	OR<br>OR	30-day mortality (in/out hospital)<br>90-day mortality (in/out hospital)	1.11 (1.06 to 1.17)<br>1.08 (1.03 to 1.13)
Aylin 2013 [16]	Elective surgical	OR<br>OR	2-day mortality (in/out hospital)<br>30-day mortality (in/out hospital)	2.67 (2.30 to 3.09)<br>1.82 (1.71 to 1.94)
McIsaac 2014 [67]	Elective surgical (non-cardiac)	OR<br>OR	2-day mortality (in/out hospital)<br>30-day mortality (in/out hospital)	2.00 (0.68 to 5.85)<br>1.96 (1.34 to 2.86)
Dubois et al 2016 [66]	Elective surgical (Friday vs Monday)	OR<br>OR<br>OR<br>OR	2-day mortality (in/out hospital)<br>In-hospital mortality<br>30-day mortality (in/out hospital)<br>90-day mortality (in/out hospital)	0.87 (0.65 to 1.16)<br>1.05 (0.93 to 1.19)<br>1.08 (0.97 to 1.21)<br>1.09 (1.00 to 1.19)
Palmer et al. 2015 [21]	Maternity	OR<br>OR	1-day neonatal mortality (in-hospital)<br>7-day perinatal mortality (in-hospital)	1.09 (1.03 to 1.15)<br>1.07 (1.02 to 1.13)

aRR: adjusted risk ratio; HR: hazard ratio; OR: odds ratio. RR: risk ratio

10.4 Different effect measures

Two studies used different effect measures for a given mortality outcome measure. The numerical estimates of the weekend effect were very similar between odds ratio and hazard ratio, and between odds ratio and risk ratio in the respective study.

Table 22 Studies in which different effect measures were used to estimate the weekend effect

**Study**	**Type of admissions**	**Outcome measure**	**Effect measure**	**Effect estimates**
Perez Concha et al. 2014 [56]	Emergency (both medical & surgical)	7-day mortality (both in and out hospital)	Odds ratio<br>Hazard ratio	1.17 (1.14 to 1.19)<br>1.16 (1.14 to 1.18)
McIsaac et al. 2014 [67]	Elective surgical (non-cardiac)	30-day mortality (both in and out hospital)	Odds ratio<br>Risk ratio	1.96 (1.34 to 2.86)<br>1.93 (1.33 to 2.79)

10.5 Multiple analyses of the same or overlapping data set(s)

This section shows that even based on the same data source, estimation of the weekend effects can still vary substantially, indicating the potentially poor signal-to-noise ratio in using the weekend effect on hospital mortality as a reliable measure of care quality.

Table 23 Comparison of weekend effect estimates made by different authors based on the same or largely overlapping datasets

Study [adequacy of statistical adjustment]	Outcome measure	Comparison	Adjusted odds ratio
**Data source: Hospital episode statistics (HES), all English hospitals, 2013-14, emergency admissions (both medical & surgical)**			
Aldridge et al. 2016 [24] [3]	In-hospital mortality	Sunday vs Wednesday	1.13 (1.10 to 1.15)
		Saturday vs Wednesday	1.09 (1.07 to 1.12)
Anselmi et al. 2016 [25] [2b]	30-day in-hospital mortality	Sunday day time vs Wednesday day time	1.061 (1.028 to 1.095)
		Sunday night time vs Wednesday day time	1.019 (0.981 to 1.058)
		Saturday day time vs Wednesday day time	1.031 (0.999 to 1.064)
		Saturday night time vs Wednesday day time	0.997 (0.960 to 1.035)
Meacock et al. 2016 [26] [2b]	30-day in-hospital mortality	Sunday vs Wednesday (admissions via A & E)	1.088 (1.063 to 1.114)
		Sunday vs Wednesday (admissions from the community)	1.278 (1.196 to 1.366)
		Saturday vs Wednesday (admissions via A & E)	1.047 (1.023 to 1.072)
		Saturday vs Wednesday (admissions from the community)	1.154 (1.082 to 1.231)
**Data source: Irish national hospital in-patient enquiry (HIPE), St James's hospital, 2002-14, emergency medical admissions***			
Conway et al. 2016 [58] [2a]	30-day in-hospital mortality	Weekend (Friday to Sunday) vs Weekday (Monday to Thursday), "multivariate logistic regression model"	1.02 (0.94 to 1.10)

		Weekend (Friday to Sunday) vs Weekday (Monday to Thursday), "Modelling mortality risk over time"	1.08 (1.01 to 1.15).
Conway et al. 2017a [59] [2a]	30-day in-hospital mortality	Weekend (Friday to Sunday) vs Weekday (Monday to Thursday), "multivariable logistic regression"	1.07 (0.98 to 1.16)
		Weekend (Friday to Sunday) vs Weekday (Monday to Thursday, "margin multivariable adjusted, incidence rate ratio"	1.03 (0.96 to 1.09)
Conway et al. 2017b [60] [4]	30-day in-hospital mortality	Weekend (Friday 17:00 to Sunday) vs weekend (Monday to Friday 17:00), "univariate adjusted odds ratio"	1.15 (1.05 to 1.24)
	30-day in-hospital mortality	Weekend (Friday 17:00 to Sunday) vs weekend (Monday to Friday 17:00), "Factoring in the time periods and the interaction between time and the weekend effect"	0.90 (0.71 to 1.12)

*Multiple estimates were reported across the three papers based on the same dataset (with exact the same number of admissions), but the effect measures and corresponding methods (e.g. type of statistical techniques/models and variables being adjusted for) were poorly described.

Appendix 11. Evidence on the weekend effect related to adverse events

Nineteen studies compared the risk of adverse events between weekend and weekday admissions. [21,29,34,35,38,40,42,43,49,50,61,66,68-70,72,75-77]

Adverse events examined include hospital acquired conditions; [35] patient safety indicators; [38,49] post-operative complications; [50] [29,34,40]; reoperation; [66] adverse maternal and neonatal outcomes [21,42,68-70,72,75,76]; re-admissions [43,61,66]; re-visit to A&E;[61] admission to intensive care unit [61,66] and clinical management incidents. [77] While an increased risk of experiencing various adverse events was observed for weekend admissions in some studies, the findings were heterogeneous and inconsistent (e.g. risk of was increased for some measures of adverse events but not increased or even decreased for other measures within individual studies; inconsistent findings with regard to the existence and magnitude of the weekend effect for a given adverse event between different studies). Findings for different measures of adverse events are presented below.

11.1 Composite measures of adverse events

In a large study using the Nationwide Inpatient Sample, Attenello and colleagues [35] found that weekend admissions are associated with a 25% increase in the odds of experiencing a hospital acquired condition, which is considered by the US Centers for Medicare & Medicaid Services as

a condition that "could reasonably have been prevented through the application of evidence–based guidelines". Falls and trauma within hospitals were the most common hospital acquired condition, which constituted of 88% of the events.

Based on data collected in a voluntary incident-reporting system, Buckley & Bulger 2012 [77] suggested that the risk of clinical management incidents (including both errors leading to harm and near misses and errors that did not cause harm) was higher among patients admitted during weekends (OR 2.738, 2.552 to 2.937). However the increased risk was most pronounced for incidents that were less serious, and no adjustment was made for severity of illness.

Table 24 Weekend effect on composite measures of adverse events reported in included studies

Composite measure of adverse events	Study	Type of admission	Adequacy of statistical adjustment	Adjusted odds ratio (95% CI)	Comment
**Hospital acquired conditions**: air embolism, retained foreign objects post surgery, poor glycaemic control, blood incompatibilities, pressure ulcers, catheter associated UTI, vascular catheter associated infection, falls/ trauma, mediastinitis post CABG, surgical site infection - after certain orthopaedic procedures, after cardiac implantable electronic device, after bariatric surgery for obesity, DVT/PE after certain orthopaedic procedure, iatrogenic pneumothorax with venous catheterisation	Attenello et al. 2015 [35]	All admissions (emergency, elective, maternity)	2b	1.25 (1.24 to 1.26)	
**Poor outcome:** death at 24 hours, cardiac arrest, ICU transfer	Khanna et al. 2011 [61]	Emergency medical admissions	2b	Not estimated as there were only 13 events	ED admission only; no difference in physician level between weekdays and weekends
**Clinical management incidents**	Buckley & Bulger 2012 [77]	Undefined admissions	4	2.738 (2.552 to 2.937)	

CABG: coronary artery bypass graft; DVT: deep vein thrombosis; PE: pulmonary embolism; ICU: intensive care unit; UTI: urinary tract infection.

11.2 Needs for further hospital care following initial admission

Four studies examined the weekend effect on various measures of readmission, reoperation, ICU admission and A&E visits following the initial admission (or following discharge from the initial admission). The results are heterogeneous: one study focusing on children [43] with inadequate adjustment of potential confounding factors reported a 9% increase in the odds for 30-day readmissions associated with weekend admissions; Khanna and colleagues, based on data from a single hospital in Chicago in which there were no difference in physician level between weekdays and weekends, found that weekend admissions were associated with significantly lower risk of ICU transfer during hospitalisation and were not associated with an increased risk of readmissions or re-visit to the A&E; [61] Dubois and colleague found an increase odds of 7% for ICU admission among elective surgeries carried out on Fridays compared with those carried out on Mondays, but no increase in 30-day re-operation or readmission was observed. [66] Walker et al. reported significantly higher risk of admitting to ICU for weekend admissions when factors available from administrative database were adjusted for, but this weekend effect was substantially attenuated when laboratory test results were also adjusted for. [13]

Table 25 Weekend effect on the need for further hospital care following initial admission reported in included studies

Further hospital care required	Study	Type of admission	Adequacy of statistical adjustment	Adjusted odds ratio (95% CI)	Comment
30-day readmission, any causes	Auger et al. 2015 [43]	All admissions	4	1.09 (1.02 to 1.18)	Children only
30-day unplanned readmission	Auger et al. 2015 [43]	All admissions	4	1.09 (1.00 to 1.18)	Children only
ICU admission	Walker et al. 2017 [13]	Emergency medical & surgical admissions	1	Results were presented in supplementary Figure 15 (a) and (b).	Significant weekend effect (adjusted relative risk around 1.20) was observed when the model adjusted for factors from administrative database; Additionally adjusting for laboratory test results significantly attenuated the weekend effect
ICU transfer during hospitalisation	Khanna et al. 2011 [61]	Emergency medical admissions	2b	0.20 (0.05 to 0.88)	ED admission only; no difference in physician level between weekdays and weekends
30 day repeat ED visit	Khanna et al. 2011 [61]	Emergency medical admissions	2b	0.95 (0.63 to 1.44)	ED admission only; no difference in physician level between weekdays and weekends
30 day repeat hospital visit/readmission	Khanna et al. 2011 [61]	Emergency medical admissions	2b	0.80 (0.51 to 1.25)	ED admission only; no difference in physician level between weekdays and weekends
30-day readmission	Dubois et al 2016 [66]	Elective surgical admissions	2b	1.02 (0.98 to 1.06)	Friday vs Monday (day of surgery)
30-day reoperation	Dubois et al 2016 [66]	Elective surgical admissions	2b	0.96 (0.90 to 1.02)	Friday vs Monday (day of surgery)

ICU admission	Dubois et al 2016 [66]	Elective surgical admissions	2b	1.07 (1.02 to 1.12)	Friday vs Monday (day of surgery)

ED: emergency department; ICU: intensive care unit

11.3 Patient safety indicators and surgical adverse events

Many studies examined the weekend effect on patient safety indicators or other measures related to adverse events during or following surgery. While an increased risk of surgical adverse events was found in several studies, the findings were not consistent across different outcomes within individual studies and were also heterogeneous across studies for a given outcome measure.

Table 26 Patient safety indicators and surgical adverse events

Outcome measures	Study	Type of admission	Adequacy of statistical adjustment	Adjusted odds ratio (95% CI)	Comment
**Peri-operative adverse events**					
Patient Safety Indicator: Complications of anaesthesia	Bendavid et al 2007 [49]	All surgical admissions (emergency, elective, maternity)	3	0.86 (0.78 to 0.95)	
Transfusion of blood products	Goldstein et al. 2014 [29]	Emergency surgical admissions	2b	1.14 (1.01 to 1.26)	Children only
Required transfusion	Zapf et al. 2015 [40]	Emergency surgical admissions	3	1.17 (0.96 to 1.43)	
Patient Safety Indicator: Retained Foreign bodies	Bendavid et al 2007 [49]	All surgical admissions (emergency,	3	0.96 (0.82 to 1.11)	

		elective, maternity)			
Accidental puncture or laceration	Goldstein et al. 2014 [29]	Emergency surgical admissions	2b	1.40 (1.14 to 1.74)	Children only
Patient Safety Indicator: Accidental cuts and lacerations during procedure, excluding maternity	Bendavid et al 2007 [49]	All surgical admissions (emergency, elective, maternity)	3	0.99 (0.95 to 1.02)	
Patient Safety Indicator: Accidental puncture or laceration	Ricciardi et al. 2016 [38]	Emergency admissions	4	0.804 (0.79 to 0.82)	
**Post-operative adverse events**					
Post-operative complications: acute kidney injury (AKI), mechanical ventilation required for >48 hours, ICU admission for >48 hours, severe sepsis, cardiovascular complications and/or the need for vasopressors for >24 hours, neurologic complications (including delirium), and wound complications (including mechanical wound complications and surgical infections)<br>One post-operative complication<br>Two post-operative complications<br>≥ three post-operative complications	Ozrazgat-Baslanti et al. 2016 [50]	All surgical admissions	3	 1.05 (0.98 to 1.13)<br>1.19 (1.09 to 1.30)<br>1.36 (1.26 to 1.47)	
30-day post-operative morbidity (1 or more complications)	Zare et al. 2007 [34]	Elective surgical admissions	2b	Could not be estimated*	Friday surgery
Haemorrhage, hematoma, or seroma	Goldstein et al. 2014 [29]	Emergency surgical admissions	2b	0.94 (0.77 to 1.15)	Children only
Patient Safety Indicator: Postoperative haemorrhage, excluding maternity	Bendavid et al 2007 [49]	All surgical admissions (emergency, elective, maternity)	3	1.07 (1.01 to 1.14)	

Patient Safety Indicator: Postoperative haemorrhage/haematoma	Ricciardi et al. 2016 [38]	Emergency admissions	4	0.966 (0.92 to 1.01)	
Dehiscence or non-healing wound	Goldstein et al. 2014 [29]	Emergency surgical admissions	2b	0.97 (0.73 to 1.27)	Children only
Patient Safety Indicator: Postoperative wound dehiscence	Ricciardi et al. 2016 [38]	Emergency admissions	4	1.355 (1.25 to 1.48)	
Wound infection or abscess	Goldstein et al. 2014 [29]	Emergency surgical admissions	2b	1.02 (0.93 to 1.12)	Children only
Developed wound complication	Zapf et al. 2015 [40]	Emergency surgical admissions	3	1.29 (1.05 to 1.58)	
Patient Safety Indicator: Postoperative hip fracture	Ricciardi et al. 2016 [38]	Emergency admissions	4	1.188 (1.03 to 1.36)	
Patient Safety Indicator: Postoperative pulmonary embolism or deep vein thrombosis	Ricciardi et al. 2016 [38]	Emergency admissions	4	1.094 (1.08 to 1.11)	
Developed sepsis following surgery	Zapf et al. 2015 [40]	Emergency surgical admissions	3	1.07 (0.84 to 1.37)	
Developed pneumonia following surgery	Zapf et al. 2015 [40]	Emergency surgical admissions	3	1.24 (1.0 to 1.54)	
Developed urinary tract infection following surgery	Zapf et al. 2015 [40]	Emergency surgical admissions	3	1.39 (1.05 to 1.85)	
**Other patient safety indicators**					
Pressure ulcer	Ricciardi et al. 2016 [38]	Emergency admissions	4	1.033 (1.02 to 1.04)	
Iatrogenic pneumothorax	Ricciardi et al. 2016 [38]	Emergency admissions	4	0.986 (0.95 to 1.03)	

Central venous catheter-related blood stream infection	Ricciardi et al. 2016 [38]	Emergency admissions	4	1.019 (1.00 to 1.04)	

*The authors stated that "Our logistic regression models for 30-day morbidity had unacceptably low c-indices (measures of predictive validity of the models) that ranged from 0.56 to 0.65, precluding a risk-adjusted assessment of postoperative morbidity in this study."

ICU: intensive care unit.

Ricciardi et al. 2016 (graded 4 for adequacy of statistical adjustment) examined mortality among patients who had experienced a patient safety indicator (PSI) event, [38] and found patients admitted during weekend had a higher odds of death following all the PSIs evaluated except central venous catheter-related blood stream infection.

Table 27 Weekend effect on death following a patient safety indicator event reported by Ricciardi et al. 2016

Patient safety indicator (PSI) related mortality	Adjusted odds ratio
Death following PSI: pressure ulcer	1.05 (1.02 to 1.08)
Death following PSI: iatrogenic pneumothorax	1.20 (1.10 to 1.31)
Death following PSI: central venous catheter-related blood stream infection	1.00 (0.92 to 1.08)
Death following PSI: postoperative hip fracture	1.27 (0.87 to 1.86)
Death following PSI: postoperative haemorrhage/haematoma	1.22 (1.06 to 1.41)
Death following PSI: postoperative pulmonary embolism or deep vein thrombosis	1.05 (1.00 to 1.11)
Death following PSI: postoperative wound dehiscence	1.12 (0.89 to 1.41)
Death following PSI: accidental puncture or laceration	1.16 (1.07 to 1.25)

11.4 Perinatal and neonatal adverse events

With a few exceptions (some of which were subgroup analyses), studies of maternity admissions generally reported relatively small or no weekend effect for perinatal and neonatal adverse events.

Table 28 Weekend effect on perinatal adverse events reported in the included studies

**Outcome measure**	**Study**	**Adequacy of statistical adjustment**	**Adjusted odds ratio (95% CI)**	**Comment**
Adverse perinatal outcome: intrapartum and early neonatal mortality, a low Apgar score, severe birth trauma (excluding cephalic haematoma, fracture of the clavicle, facial nerve injury and injury to the brachial plexus), and admission to a NICU on the same or the day after birth	Gijsen et al. 2012 [70]	2b	1.01 (0.97 to 1.05)	Combined results using data from 7 reported subgroups
Perinatal adverse outcome: intrapartum or early neonatal death (number of deaths within 7 days after live birth), 5-minute Apgar score below 7, or transfer of the newborn to a neonatal intensive care unit after birth	de Graaf et al. 2010 [68]	2b		
Tertiary hospitals, Saturday vs Tuesday			1.16 (1.05 to 1.30)	
Tertiary hospitals, Sunday vs Tuesday			1.02 (0.91 to 1.13)	
Non-tertiary hospitals, Saturday vs Tuesday			1.01 (0.93 to 1.08)	
Non-tertiary hospitals, Sunday vs Tuesday			1.03 (0.95 to 1.11)	
Composite neonatal adverse outcomes: birth trauma, neonatal seizures (defined using ICD-9 codes), 5-min Apgar score <7, admission to the NICU, neonatal death (all defined using vital statistics data)	Snowden et al. 2016 [42]	4	1.04 (1.01 to 1.06)	Both high & low volume days
Birth asphyxia	Snowden et al. 2013 [75]	4	1.27 (1.07 to 1.51)	Suggested the weekend effect was stronger on high-volume days* in which there was a lower rate of caesarean delivery
Apgar score 0-6	Gijsen et al. 2012 [70]	2b	1.00 (0.94 to 1.06)	Combined results using data from 7 reported subgroups

5 minute Apgar score <7	Frank-Wolf et al. 2016 [69]	4	p=0.118	
Apagr score <7	Snowden et al. 2016 [42]	4	1.14 (1.06 to 1.23)	Both high & low volume days
Cord pH <7	Frank-Wolf et al. 2016 [69]	4	p=0.514	
Injury to neonate	Palmer et al. 2015 [21]	2b	1.06 (1.02 to 1.09)	
Birth trauma (liveborn, patient Safety Indicator): all newborn babies (excluding subdural or cerebral haemorrhage, preterm infants, skeletal injury, osteogenesis imperfecta)	Bendavid et al 2007 [49]	3	1.06 (1.03 to 1.10)	
Neonatal birth trauma	Snowden et al. 2016 [42]	4	0.99 (0.95 to 1.03)	Both high & low volume days
Selected neonatal infections	Palmer et al. 2015 [21]	2b	1.01 (0.98 to 1.04)	
Neonatal encephalopathy	Wu et al. 2011 [76]	4	Risk ratio 0.96 (0.86 to 1.06)	Unadjusted; variable not included in multivariate analysis
Neonatal seizures	Snowden et al. 2016 [42]	4	1.04 (0.81 to 1.32)	Both high & low volume days
NICU admission	Snowden et al. 2016 [42]	4	1.07 (1.04 to 1.11)	Both high & low volume days
Three day neonatal readmissions	Palmer et al. 2015 [21]	2b	1.04 (1.00 to 1.08)	

*Defined as days in which the number of births exceeded the hospital's 75th percentile for daily births for each individual hospital.

CI: confidence interval; ICD-9: International Classification of Diseases, 9th revision; NICU: neonatal intensive care unit

11.5 Maternal adverse events

Weekend effect was found for several adverse events in some studies, although the statistical adjustment was only judged to be partial or inadequate in many cases.

Table 29 Weekend effect on maternal adverse events reported in the included studies

Outcome measure	Study	Adequacy of statistical adjustment	Adjusted odds ratio (95% CI)
Perineal tear	Palmer et al. 2015 [21]	2b	1.00 (0.98 to 1.03)
Severe perineal laceration	Snowden et al. 2016 [42]	4	1.08 (1.04 to 1.12)
Obstetric trauma during Vaginal delivery with instrumentation (patient safety indicator)	Bendavid et al 2007 [49]	3	1.00 (0.98 to 1.02)
Obstetric trauma during vaginal delivery without instrumentation (patient safety indicator)	Bendavid et al 2007 [49]	3	1.03 (1.02 to 1.04)
Obstetric trauma during Caesarean section (patient safety indicator)	Bendavid et al 2007 [49]	3	1.36 (1.29 to1.44)
Composite maternal adverse events: obstetric infection (chorioamnionitis, endometritis and wound infection subsequent to caesarean delivery), haemorrhage (a composite of post-partum haemorrhage diagnosis codes and maternal blood transfusion procedure codes), severe perineal lacerations (third or fourth degree), prolonged maternal length of stay (LOS; LOS >3 days for vaginal deliveries and >5 days for caesarean deliveries)	Snowden et al. 2016 [42]	4	1.12 (1.10 to 1.15)
Pelvic floor morbidity: episiotomy, third- or fourth degree laceration, and vulvar or perineal hematoma or other trauma	Lyndon et al. 2015 [72]	4	1.01 (0.99 to 1.03)
Severe morbidity: hysterectomy, unplanned return to operating room, transfer to intensive care unit, maternal death, or length of stay >= 90th percentile for mode of birth with a diagnosis of severe postpartum hemorrhage, maternal sepsis, deep vein thrombosis, pulmonary embolism, uterine rupture, respiratory failure, heart failure, puerperal cerebrovascular accident, severe anaesthetic complication, maternal shock, disseminated intravascular coagulation, or renal failure	Lyndon et al. 2015 [72]	4	1.03 (0.94 to 1.13)
Maternal Haemorrhage	Snowden et al. 2016 [42]	4	1.05 (1.01 to 1.08)
Puerperal infection	Palmer et al. 2015 [21]	2b	1.06 (1.01 to 1.11)
Obstetric infection	Snowden et al. 2016 [42]	4	1.09 (1.05 to 1.13)

Maternal Prolonged length of stay (>3days for vaginal delivery, >5 days for caesarean section)	Snowden et al. 2016 [42]	4	1.21 (1.17 to 1.25)
Three day maternal readmissions	Palmer et al. 2015 [21]	2b	0.93 (0.84 to 1.02)

LOS: length of stay

Appendix 12. Evidence on the weekend effect related to length of stay (LOS)

Fifteen studies compared hospital LOS between weekend and weekday admissions. [8,15,23,28,29,39,40,42,45,59-64,66] Data reported in individual studies are shown in Table 30 below. The majority of studies show that the (unadjusted) mean or median hospital LOS was shorter (by one day or less in most cases) for admissions during weekends compared with admissions during weekdays. There are a few notable exceptions in which longer LOS associated with weekend admissions was observed: a study (which covered all admissions including maternity admissions) by Freemantle and colleagues reported a median LOS of 3 days for weekend admissions compared with a median LOS of 1 day for weekday admissions;[23] two studies of elective admissions [15,66] also reported a longer median and/or mean LOS for weekend admissions. A further study reported an adjusted odds ratio of 1.21 (95% CI 1.17 to 1.25) prolonged LOS (defined as > 3 days for vaginal deliveries and >5 days for caesarean deliveries) for maternity admissions over the weekend, and this increase was more pronounced for weekend days in which hospitals experienced high birth volumes.[42]. Finally, Earnest reported an adjusted difference of 0.31 days (longer for weekend admissions) after age, sex, admission type (emergency or elective) and source of admission (ED, ward, outpatients) were taken into account.[45]

Table 30 Length of hospital stay for weekend and weekday admissions

Study	Weekend definition and LOS	Weekday definition and LOS	Measure of weekend-weekday differences
Earnest et al. 2006 [45]<br>All admissions	Weekend (Friday to Sunday)	Weekday (Monday to Thursday)	Adjusted difference 0.31 days (longer for weekend)<br>p<0.001
Earnest et al. 2006 [45]<br>All admissions	Eve of public holiday	Weekday (Monday to Thursday)	Adjusted difference 0.71 days (longer for public holiday)<br>p<0.001
Mikulich et al. 2011 [62]<br>All admissions	Weekends<br>n= 5355<br>mean (SD): 6.3 (6.4)	Weekdays<br>n= 20478<br>mean (SD): 6.9 (6.7)	NR
Freemantle et al. 2015 [23]<br>All admissions including maternity	Saturday<br>n= NR<br>median (IQR): 3 (2-5)	Monday to Friday<br>n= NR<br>median (IQR): 1 (1-3)	NR
Freemantle et al. 2015 [23]<br>All admissions including maternity	Sunday<br>n= NR<br>median (IQR): 3 (2-6)	Monday to Friday<br>n= NR<br>median (IQR): 1 (1-3)	NR
Freemantle et al. 2015 [23]<br>All admissions, patients who died in hospital	Saturday & Sunday<br>n= NR<br>median (IQR): 8 (4-17)	Monday to Thursday<br>n= NR<br>median 9; (IQR 4-18 for Monday, 5-18 for Tuesday, 4-19 for Wednesday & Thursday)	NR
Freemantle et al. 2015 [23]<br>All admissions, patients in the highest risk category	Saturday<br>n= NR<br>median (IQR): 5 (3-12)<br><br>Sunday<br>n= NR<br>median (IQR): 6 (3-12)	Monday to Friday<br>n= NR<br>median (IQR): 4 for Monday to Friday except Thursday; (IQR Monday 2-10, Tuesday to Friday 2-9)	NR
Freemantle et al. 2015 [23]	Saturday & Sunday<br>n= NR<br>median (IQR): 8 (4-17)	Monday to Friday<br>n= NR	NR

All admissions, patients in the highest risk category who died in hospital		median (IQR): 9 (IQR 5-18 Monday, Wednesday & Thursday,<br>5-19 Tuesday and Friday)	
Mohammed et al. 2012 [15]<br>Emergency admissions	Weekends<br>n= 735933<br>median (IQR): 3 (8)	Weekdays<br>n= 2369316<br>median (IQR): 4 (8)	All patients
Mohammed et al. 2012 [15]<br>Emergency admissions	Weekends<br>n= 684011<br>median (IQR): 3 (8)	Weekdays<br>n= 2214555<br>median (IQR): 3 (7)	Subgroup: patients discharged alive
Mohammed et al. 2012 [15]<br>Emergency admissions	Weekends<br>n= 51922<br>median (IQR): 6 (15)	Weekdays<br>n= 154761<br>median (IQR): 8 (17)	Subgroup: patients who died in hospital
Mohammed et al. 2017 [28]<br>Emergency admissions	Weekends<br>n= 11332<br>mean (SD): 7.34 (11.61)	Weekdays<br>n= 35785<br>mean (SD): 7.54 (11.55)	NR
Conway et al. 2017a [59]<br>Emergency medical admissions	Weekends<br>n= 27487<br>median (IQR): 5.0 (2.7 to 10.1)	Weekdays<br>n= 46402<br>median (IQR): 5.1 (1.9 to 9.4)	NR
Conway et al. 2017b [60]<br>Emergency medical admissions (discharged or deceased by day 28)	Weekends<br>n= 27330<br>median (IQR): 4.9 (2.7 to 10.0)	Weekdays<br>n= 45867<br>median (IQR): 5.1 (1.9 to 9.1)	NR
Conway et al. 2017b [60]<br>Emergency medical admissions (length of stay between 28 to 90 days)	Weekends<br>n= 2406<br>median (IQR): 41.7 (33.6 to 57.5)	Weekdays<br>n= 4333<br>median (IQR): 42.6 (34.0 to 57.0)	NR
Khanna et al. 2011 [61]<br>Emergency medical admissions	Weekend<br>n= 183<br>median (IQR): 3.8 (NR)	Weekday<br>n= 641<br>median (IQR): 4.3 (NR)	NR
Vest-Hansen et al. 2015 [63]<br>Emergency medical admissions	Weekend<br>Daytime<br>n= 29140<br>Median (IQR): 3 (1-7)	Weekday<br>Office hour<br>n= 87764<br>Median (IQR): 3 (1-7)	NR

	Night time<br>n= 13976<br>Median (IQR): 3 (1-6)	Out of hour<br>n= 43312<br>Median (IQR): 2 (1-7)	
Beecher et al. 2015 [64]<br>Emergency surgical admissions	Weekend admissions, 3 days (median)	Weekday admissions, 4 days (median)	p=0.017
Goldstein et al. 2014 [29]<br>Emergency surgical admissions	NR	NR	Mentioned length of stay in the Methods but did not report results
McCallum et al. 2016 [8]<br>Emergency surgical admissions	Saturday<br>n= 40617<br>mean 5.81 (95% CI 5.70 to 5.92)<br><br>Sunday<br>n= 40474<br>mean 6.02 (95% CI 5.91 to 6.14)	Wednesday<br>n= 56955<br>mean 6.15 (95% CI 6.05 to 6.25)	By day of admission
McCallum et al. 2016 [8]<br>Emergency surgical admissions	Saturday<br>n= 7159<br>mean 7.91 (95% CI 7.62 to 8.20)<br><br>Sunday<br>n= 6052<br>mean 7.47 (95% CI 7.17 to 7.77)	Wednesday<br>n= 10633<br>mean 8.71 (95% CI 8.45 to 8.97)	By day of surgery
Zapf et al. 2015 [40]<br>Emergency surgical admissions	Weekend<br>n= 19078<br>Mean (SD): 3 (NR)	Weekday<br>n= 61783<br>Mean (SD): 3 (NR)	Coefficient 0.011, 95% CI -0.01 to 0.032, p >0.05
Mohammed et al. 2012 [15]<br>Elective admissions	Weekends<br>n= 127562<br>median (IQR): 3 (3)	Weekdays<br>n= 1407705<br>median (IQR): 1 (3)	All patients
Mohammed et al. 2012 [15]<br>Elective admissions	Weekends<br>n= 126576<br>median (IQR): 3 (5)	Weekdays<br>n= 1400429<br>median (IQR): 1 (3)	Subgroup: patients discharged alive
Mohammed et al. 2012 [15]<br>Elective admissions	Weekends<br>n= 986	Weekdays<br>n= 7276	Subgroup: patients who died in hospital

	median (IQR): 12 (21)	median (IQR): 12 (20)	
Dubois et al. 2016 [66]<br>Elective surgical admissions	Friday<br>n= 65139<br>mean (SD): 7.9 (8.8)<br>median (IQR): 6 (5-8)	Monday<br>n= 77082<br>mean (SD): 7.5 (9.1)<br>median (IQR): 5 (4-8)	NR
Snowden et al. 2016 [42]<br>Maternity admissions<br><br>Overall<br><br><br>Low/average-volume day<br><br><br>High volume day (days in which the number of births exceeded each hospital's own 75th percentile for daily births)	Weekends<br><br><br>n= 177233<br>4.2%<br><br>n= 153287<br>4.1%<br><br>n= 23946<br>4.7% (p<0.001 vs low/average-volume day)	Weekdays<br>Overall<br><br>n= 547744<br>3.5%<br><br>n= 345097<br>3.5%<br><br>n=202647<br>3.5% (p=0.161 vs low/average-volume day)	Prolonged length of stay (> 3 days for vaginal deliveries and >5 days for caesarean deliveries)<br>p<0.001<br>Adjusted OR 1.21 (1.17 to 1.25)

CI: confidence interval; IQR: interquartile range; NR: not reported; OR: odds ratio; SD: standard deviation

Appendix 13. Evidence on the weekend effect related to patient satisfaction

Only one study compared quantitative measures of patient satisfaction between weekend and weekday admissions.[46] Based on data from the 2014 NHS adult inpatient survey (154 trusts, with 59,083 respondents representing a 47% response rate) and accident and emergency (A&E) department surveys (142 trusts, with 39,320 respondents representing a 34% response rate) and the adult inpatient survey, Graham compared the reported satisfaction of patients who attended A&E departments, admitted to hospital or discharged from hospital at weekends (including public holidays) with those who experienced these events during weekdays. Patients who died following the A&E visits/admissions were excluded from the surveys. Patients admitted at weekends were less likely to respond compared to those admitted during weekdays, but this was accounted for by patient and admission characteristics (e.g. age groups, emergency vs elective admissions and ethnicity).

The findings, which adjusted for patient age group, sex, ethnicity, use of proxy response (self-completed or supported), limiting long-term conditions, NHS trust, route of admission (emergency or planned, for the inpatient survey only) and destination post discharge (admitted or discharged, for the A&E survey only), show that patients who attended A&E at weekends were significantly more satisfied about 'doctors and nurses' and 'care and treatment' compared with those who attended during weekdays. Patients admitted to hospital via A&E at weekends were also more positive about the information given to them in A&E. There were no significant differences in other dimensions of care covered in the surveys.[46]

*Table 31 Data from NHS inpatient survey reported in Graham 2017, adjusted mean values**

	**Weekend**	**Weekday**	**Difference (positive value favours weekend)**	**p value**
**Admissions (n)**	10382	48701	-	-
How much information was given to you?	8.160	7.891	0.269	<0.001
Were you given enough privacy?	8.358	8.277	0.081	0.123
Did you feel that you had to wait a long time?	6.849	6.786	0.063	0.338
**Discharges (n)**	11525	47558		
Information on discharge	6.321	6.409	−0.088	0.113
Medicines information on discharge	7.085	7.136	−0.051	0.373

*Out of a scale between 0 to 10 for each items in the survey

Appendix 14. GRADE assessment for overall quality of evidence

Table 32 Justification for GRADE assessment of overall quality of evidence for mortality

Domain	Upgrade/downgrade	Justification
Baseline rating	Low	All included studies were observational studies
1. Risk of Bias	Downgrade one level	Key potential confounding factors (urgency/severity) were adjusted for only in a small number of studies with relatively small sample sizes.
2. Inconsistency	None (could have been downgraded)	There is notable heterogeneity between estimates within studies and between studies, and the 95% credible intervals for I^2 cannot rule out a very high level of heterogeneity (see Table 2 of the main text). However as the rating is already downgraded to "very low", no further downgrading is possible.
3. Indirectness	None (could have been downgraded)	Timing of the admissions was used as a proxy for hospital care quality. Therefore inference was made indirectly. As the rating is already downgraded to "very low", no further downgrading is possible.
4. Imprecision	None	This is not a particular concern given the large volume of evidence included. The 95% credible interval (1.10 to 1.23) is reasonably narrow.
5. Publication Bias	None	Although funnel plot asymmetry was observed, our sensitivity analysis using data augmentation methods showed that adjustment for the asymmetry only had a small impact on the pooled estimate.
6. Large magnitude of effect	None	The magnitude of effect was not large and could plausibly be attributed to confounding.
7. Dose response	None	Limited evidence from two studies examining the relationship between staffing level/7-day service provision and mortality did not show correlation between them. [24,27]
8. Effect of plausible confounding factors	None	Plausible confounding factors would produce an effect in the same direction as the observed weekend effect
Final rating	Very low	This is in relation to using the estimated weekend effect on mortality to infer weekday/weekend difference in care quality in the hospital.

Table 33 Justification for GRADE assessment of overall quality of evidence for adverse events

Domain	Upgrade/downgrade	Justification
Baseline rating	Low	All included studies were observational studies
1. Risk of Bias	Downgrade one level	Potential confounding factors were inadequately adjusted or not adjusted at all among included studies.
2. Inconsistency	None (could have been downgraded)	Inconsistency was observed between studies and between different adverse events within studies. However as the rating is already downgraded to “very low”, no further downgrading is possible.
3. Indirectness	None (could have been downgraded)	Timing of the admissions was used as a proxy for hospital care quality. Therefore inference was made indirectly. In addition, measures such as hospital acquired conditions and patient safety indicators are proxy measures of adverse events arising from suboptimal care. There is therefore some level of indirectness in the evidence. However as the rating is already downgraded to “very low”, no further downgrading is possible.
4. Imprecision	None	The level of precision varied by individual adverse events. As the rating is already downgraded to “very low”, no further downgrading is required.
5. Publication Bias	None	We were unable to assess publication bias due to lack of study registry, and we did not carry out meta-analyses or construct funnel plots for this outcome given the diverse measures used.
6. Large magnitude of effect	None	The magnitude of effect was not large and was inconsistent, and could plausibly be attributed to confounding.
7. Dose response	None	Limited evidence from one study [61] showed no difference in physician level between weekday and weekend.
8. Effect of plausible confounding factors	None	Plausible confounding factors would produce an effect in the same direction as the observed weekend effect.
Final rating	Very low	This is in relation to using the estimated weekend effect on adverse events to infer weekday/weekend difference in care quality in the hospital.

Table 34 Justification for GRADE assessment of overall quality of evidence for length of hospital stay

**Domain**	**Upgrade/downgrade**	**Justification**
Baseline rating	Low	All included studies were observational studies
1. Risk of Bias	Downgrade one level	Potential confounding factors were either inadequately adjusted not adjusted at all in the included studies.
2. Inconsistency	None (could have been downgraded)	There is notable heterogeneity between studies, not just in the magnitude but in the direction (i.e. some found the length of stay for weekend admissions was longer than weekday admissions while others found the opposite). However as the rating is already downgraded to "very low", no further downgrading is possible.
3. Indirectness	None (could have been downgraded)	Timing of the admissions was used as a proxy for hospital care quality. Therefore inference was made indirectly. Length of stay was directly measured from administrative records.
4. Imprecision	None	This is not a particular concern given the large number of admissions examined in the included studies.
5. Publication Bias	None	We were unable to assess publication bias due to lack of study registry, and we did not carry out meta-analyses or construct funnel plots for this outcome. Publication bias may not be a major concern given the diverse findings reported in the included studies.
6. Large magnitude of effect	None	The magnitude of effect was not large and was inconsistent.
7. Dose response	None	Evidence was lacking to allow assessment of dose response.
8. Effect of plausible confounding factors	None	Different confounding factors may produce effects in the same or opposite direction as the weekend effect (where observed).
Final rating	Very low	This is in relation to using the estimated differences in the length of stay to infer weekday/weekend difference in care quality in the hospital.

Table 35 Justification for GRADE assessment of overall quality of evidence for patient satisfaction

Domain	Upgrade/downgrade	Justification
Baseline rating	Low	All included studies were observational studies
1. Risk of Bias	Downgrade one level	Only one study provided data.[46] Adjustment for potential confounding factors was very limited.
2. Inconsistency	None	We were unable to assess this domain as only one study provided data for this outcome.[46]
3. Indirectness	None (could have been downgraded)	Timing of the admissions was used as a proxy for hospital care quality. Only one study provided data on this outcome,[46] and the analysis of inpatient survey had to focus on questions related to admission and discharge processes rather than the period of stay as inpatient.
4. Imprecision	None	The sample size was reasonably large, although only one study provided data on this outcome.
5. Publication Bias	None	Given that only one study was found and that there is no study registry available, we were unable to assess the potential impact of publication bias.
6. Large magnitude of effect	None	The magnitude of effect was not large.
7. Dose response	None	Evidence was lacking to allow assessment of dose response.
8. Effect of plausible confounding factors	None	Different confounding factors may produce effects in the same or opposite direction as the weekend effect (where observed).
Final rating	Very low	This is in relation to using the estimated differences in patient satisfaction between weekday and weekend admissions to infer weekday/weekend differences in care quality in the hospital.

References for appendices

1. Wells GA, Shea B, O'Connell D, et al. The Newcastle-Ottawa Scale (NOS) for assessing the quality of nonrandomised studies in meta-analyses. http://www.ohri.ca/programs/clinical_epidemiology/oxford.asp Accessed 13 February 2014.
2. Chen YF, Boyal A, Sutton E, et al. The magnitude and mechanisms of the weekend effect in hospital admissions: A protocol for a mixed methods review incorporating a systematic review and framework synthesis. *Syst Rev* 2016;**5**:84.
3. Sutton AJ, Abrams KR. Bayesian methods in meta-analysis and evidence synthesis. *Statistical methods in medical research* 2001;**10**(4):277-303.
4. Gelman A, Jakulin A, Pittau MG, Su Y-S. A weakly informative default prior distribution for logistic and other regression models. *Ann Applied Stat* 2008;**2**(4):1360-83.
5. Turner RM, Jackson D, Wei Y, Thompson SG, Higgins JPT. Predictive distributions for between-study heterogeneity and simple methods for their application in Bayesian meta-analysis. *Stat Med* 2015;**34**(6):984-98.
6. Higgins JP, Thompson SG, Deeks JJ, Altman DG. Measuring inconsistency in meta-analyses. *BMJ* 2003;**327**(7414):557-60.
7. Carpenter B, Gelman A, Hoffman MD, et al. Stan: A probabilistic programming language. *J Stat Softw* 2017;**76**(1):DOI: 10.18637/jss.v076.i01.
8. McCallum IJ, McLean RC, Dixon S, O'Loughlin P. Retrospective analysis of 30-day mortality for emergency general surgery admissions evaluating the weekend effect. *Br J Surg* 2016;**103**(11):1557-65.
9. Roberts SE, Thorne K, Akbari A, Samuel DG, Williams JG. Weekend emergency admissions and mortality in England and Wales. *Lancet* 2015;**385**(9980):1829.
10. Han L, Sutton M, Clough S, Warner R, Doran T. Impact of out-of-hours admission on patient mortality: longitudinal analysis in a tertiary acute hospital. *BMJ Qual Saf* 2017;**29**:29.
11. Aylin P, Yunus A, Bottle A, Majeed A, Bell D. Weekend mortality for emergency admissions. A large, multicentre study. *Quality & Safety in Health Care* 2010;**19**(3):213-7.
12. Ozdemir BA, Sinha S, Karthikesalingam A, et al. Mortality of emergency general surgical patients and associations with hospital structures and processes. *Br J Anaesth* 2016;**116**(1):54-62.
13. Walker AS, Mason A, Quan TP, et al. Mortality risks associated with emergency admissions during weekends and public holidays: an analysis of electronic health records. *Lancet* 2017;**390**(10089):62-72.
14. Maggs F, Mallet M. Mortality in out-of-hours emergency medical admissions--more than just a weekend effect. *J R Coll Physicians Edinb* 2010;**40**(2):115-8.
15. Mohammed MA, Sidhu KS, Rudge G, Stevens AJ. Weekend admission to hospital has a higher risk of death in the elective setting than in the emergency setting: a retrospective database study of national health service hospitals in England. *BMC Health Serv Res* 2012;**12**:87.
16. Aylin P, Alexandrescu R, Jen MH, Mayer EK, Bottle A. Day of week of procedure and 30 day mortality for elective surgery: retrospective analysis of hospital episode statistics. *BMJ* 2013;**346**:f2424.
17. Ruiz M, Bottle A, Aylin PP. Exploring the impact of consultants' experience on hospital mortality by day of the week: a retrospective analysis of hospital episode statistics *BMJ Qual Saf* 2016;**26**:337–44.
18. Freemantle N, Richardson M, Wood J, et al. Weekend hospitalization and additional risk of death: an analysis of inpatient data. *J R Soc Med* 2012;**105**(2):74-84.
19. Ruiz M, Bottle A, Aylin PP. The Global Comparators project: international comparison of 30-day in-hospital mortality by day of the week. *BMJ Qual Saf* 2015.

20. Meacock R, Doran T, Sutton M. What are the Costs and Benefits of Providing Comprehensive Seven-day Services for Emergency Hospital Admissions? *Health Economics* 2015;**24**(8):907-12.
21. Palmer WL, Bottle A, Aylin P. Association between day of delivery and obstetric outcomes: observational study. *BMJ* 2015;**351**:h5774.
22. Shiue I, McMeekin P, Price C. Retrospective observational study of emergency admission, readmission and the 'weekend effect'. *BMJ Open* 2017;**7**(3):e012493.
23. Freemantle N, Ray D, McNulty D, et al. Increased mortality associated with weekend hospital admission: a case for expanded seven day services? [Erratum appears in BMJ. 2016;352:i1762; PMID: 27025883]. *BMJ* 2015;**351**:h4596.
24. Aldridge C, Bion J, Boyal A, et al. Weekend specialist intensity and admission mortality in acute hospital trusts in England: a cross-sectional study. *Lancet* 2016;**388**(10040):178-86.
25. Anselmi L, Meacock R, Kristensen SR, Doran T, Sutton M. Arrival by ambulance explains variation in mortality by time of admission: retrospective study of admissions to hospital following emergency department attendance in England. *BMJ Qual Saf* 2016:Published online first, doi:10.1136/bmjqs-2016-005680.
26. Meacock R, Anselmi L, Kristensen SR, Doran T, Sutton M. Higher mortality rates amongst emergency patients admitted to hospital at weekends reflect a lower probability of admission. *J Health Serv Res Policy* 2016:published online first, doi:10.1177/1355819616649630.
27. Meacock R, Sutton M. Elevated mortality among weekend hospital admissions is not associated with adoption of seven day clinical standards. *Emerg Med J* 2017;**08**:08.
28. Mohammed M, Faisal M, Richardson D, et al. Impact of the level of sickness on higher mortality in emergency medical admissions to hospital at weekends. *J Health Serv Res Policy* 2017:Published online first, doi: 10.1177/1355819617720955.
29. Goldstein SD, Papandria DJ, Aboagye J, et al. The "weekend effect" in pediatric surgery - increased mortality for children undergoing urgent surgery during the weekend. *J Pediatr Surg* 2014;**49**(7):1087-91.
30. Gordon HS, Johnson ML, Wray NP, et al. Mortality after noncardiac surgery: prediction from administrative versus clinical data. *Med Care* 2005;**43**(2):159-67.
31. Gould JB, Qin C, Marks AR, Chavez G. Neonatal mortality in weekend vs weekday births. *JAMA* 2003;**289**(22):2958-62.
32. Cram P, Hillis SL, Barnett M, Rosenthal GE. Effects of weekend admission and hospital teaching status on in-hospital mortality. *Am J Med* 2004;**117**(3):151-7.
33. Hamilton P, Restrepo E. Sociodemographic factors associated with weekend birth and increased risk of neonatal mortality. *J Obstet Gynecol Neonatal Nurs* 2006;**35**(2):208-14.
34. Zare MM, Itani KM, Schifftner TL, Henderson WG, Khuri SF. Mortality after nonemergent major surgery performed on Friday versus Monday through Wednesday. *Ann Surg* 2007;**246**(5):866-74.
35. Attenello FJ, Wen T, Cen SY, et al. Incidence of "never events" among weekend admissions versus weekday admissions to US hospitals: national analysis. *BMJ* 2015;**350**:h1460.
36. Ricciardi R, Roberts PL, Read TE, Baxter NN, Marcello PW, Schoetz DJ. Mortality rate after nonelective hospital admission. *Arch Surg* 2011;**146**(5):545-51.
37. Ricciardi R, Nelson J, Roberts PL, Marcello PW, Read TE, Schoetz DJ. Is the presence of medical trainees associated with increased mortality with weekend admission? *BMC Medical Education* 2014;**14**:4.
38. Ricciardi R, Nelson J, Francone TD, et al. Do patient safety indicators explain increased weekend mortality? *J Surg Res* 2016;**200**(1):164-70.
39. An R. Impact of weekend admission on in-hospital mortality among U.S. adults, 2003-2013. *Ann Epidemiol* 2017;**27**(12):790-95.

40. Zapf MA, Kothari AN, Markossian T, et al. The "weekend effect" in urgent general operative procedures. *Surgery* 2015;**158**(2):508-14.
41. Sharp AL, Choi H, Hayward RA. Don't get sick on the weekend: an evaluation of the weekend effect on mortality for patients visiting US EDs. *Am J Emerg Med* 2013;**31**(5):835-7.
42. Snowden JM, Kozhimannil KB, Muoto I, Caughey AB, McConnell KJ. A 'busy day' effect on perinatal complications of delivery on weekends: a retrospective cohort study. *BMJ Qual Saf* 2016;**29**:29.
43. Auger KA, Davis MM. Pediatric weekend admission and increased unplanned readmission rates. *J Hosp Med* 2015;**10**(11):743-5.
44. Coiera E, Wang Y, Magrabi F, Concha OP, Gallego B, Runciman W. Predicting the cumulative risk of death during hospitalization by modeling weekend, weekday and diurnal mortality risks. *BMC Health Serv Res* 2014;**14**:226.
45. Earnest A, Chen MI, Seow E. Exploring if day and time of admission is associated with average length of stay among inpatients from a tertiary hospital in Singapore: an analytic study based on routine admission data. *BMC Health Serv Res* 2006;**6**:6.
46. Graham C. People's experiences of hospital care on the weekend: secondary analysis of data from two national patient surveys. *BMJ Qual Saf* 2017;**29**:29.
47. Lee KG, Vaithilingam I. A study of weekend and off-hour effect on mortality in a public hospital in Malaysia. *Medical Journal of Malaysia* 2012;**67**(5):478-82.
48. Madsen F, Ladelund S, Linneberg A. High levels of bed occupancy associated with increased inpatient and thirty-day hospital mortality in Denmark. *Health Affairs* 2014;**33**(7):1236-44.
49. Bendavid E, Kaganova Y, Needleman J, Gruenberg L, Weissman JS. Complication rates on weekends and weekdays in US hospitals. *Am J Med* 2007;**120**(5):422-8.
50. Ozrazgat-Baslanti T, Blanc P, Thottakkara P, et al. Preoperative assessment of the risk for multiple complications after surgery. *Surgery* 2016;**160**(2):463-72.
51. Barba R, Losa JE, Velasco M, Guijarro C, Garcia de Casasola G, Zapatero A. Mortality among adult patients admitted to the hospital on weekends. *Eur J Intern Med* 2006;**17**(5):322-4.
52. Bell CM, Redelmeier DA. Mortality among patients admitted to hospitals on weekends as compared with weekdays. *N Engl J Med* 2001;**345**(9):663-8.
53. De Giorgi A, Fabbian F, Tiseo R, et al. Weekend hospitalization and inhospital mortality: a gender effect? *Am J Emerg Med* 2015;**33**(11):1701-3.
54. Handel A, Patel SV, Skingsley A, Bramley K, Sobieski R, Ramagopalan SV. Weekend admissions as an independent predictor of mortality: An analysis of Scottish hospital admissions. *BMJ Open* 2012;**2**(6):e001789.
55. Lee LH, Hsueh YS, Yang CH. The relationship between admission on weekends or holidays and treatment outcome. [Chinese]. *Taiwan Journal of Public Health* 2006;**25**(2):107-14.
56. Perez Concha O, Gallego B, Hillman K, Delaney GP, Coiera E. Do variations in hospital mortality patterns after weekend admission reflect reduced quality of care or different patient cohorts? A population-based study. *BMJ Qual Saf* 2014;**23**:215-22.
57. Sullivan C, Staib A, Eley R, et al. Who is less likely to die in association with improved National Emergency Access Target (NEAT) compliance for emergency admissions in a tertiary referral hospital? *Aust Health Rev* 2016;**40**(2):149-54.
58. Conway R, Cournane S, Byrne D, O'Riordan D, Silke B. Time patterns in mortality after an emergency medical admission; relationship to weekday or weekend admission. *Eur J Intern Med* 2016;**18**:18.
59. Conway R, Cournane S, Byrne D, O'Riordan D, Silke B. Improved mortality outcomes over time for weekend emergency medical admissions. *Ir J Med Sci* 2017;**11**:11.
60. Conway R, Cournane S, Byrne D, O'Riordan D, Silke B. Survival analysis of weekend emergency medical admissions. *QJM* 2017;**110**(5):291-97.

61. Khanna R, Wachsberg K, Marouni A, Feinglass J, Williams MV, Wayne DB. The association between night or weekend admission and hospitalization-relevant patient outcomes. *J Hosp Med* 2011;**6**(1):10-4.
62. Mikulich O, Callaly E, Bennett K, O'Riordan D, Silke B. The increased mortality associated with a weekend emergency admission is due to increased illness severity and altered case-mix. *Acute Med* 2011;**10**(4):182-7.
63. Vest-Hansen B, Riis AH, Sorensen HT, Christiansen CF. Out-of-hours and weekend admissions to Danish medical departments: admission rates and 30-day mortality for 20 common medical conditions. *BMJ Open* 2015;**5**(3):e006731.
64. Beecher S, O'Leary DP, McLaughlin R. Increased risk environment for emergency general surgery in the context of regionalization and specialization. *Int J Surg* 2015;**21**:112-4.
65. Gillies MA, Lone NI, Pearse RM, et al. Effect of day of the week on short- and long-term mortality after emergency general surgery. *Br J Surg* 2017;**104**(7):936-45.
66. Dubois L, Vogt K, Vinden C, et al. Association between day of the week of elective surgery and postoperative mortality. *CMAJ* 2016;**17**:17.
67. McIsaac DI, Bryson GL, van Walraven C. Elective, major noncardiac surgery on the weekend: a population-based cohort study of 30-day mortality. *Med Care* 2014;**52**(6):557-64.
68. de Graaf JP, Ravelli AC, Visser GH, et al. Increased adverse perinatal outcome of hospital delivery at night. *BJOG* 2010;**117**(9):1098-107.
69. Frank-Wolf M, Tovbin J, Wiener Y, Neeman O, Kurzweil Y, Maymon R. Is there a correlation between time of delivery and newborn cord pH? *J Matern Fetal Neonatal Med* 2016:1-4.
70. Gijsen R, Hukkelhoven CW, Schipper CM, Ogbu UC, de Bruin-Kooistra M, Westert GP. Effects of hospital delivery during off-hours on perinatal outcome in several subgroups: a retrospective cohort study. *BMC Pregnancy Childbirth* 2012;**12**:92.
71. Luo ZC, Liu S, Wilkins R, Kramer MS. Risks of stillbirth and early neonatal death by day of week. *Can Med Assoc J* 2004;**170**(3):337-41.
72. Lyndon A, Lee HC, Gay C, Gilbert WM, Gould JB, Lee KA. Effect of time of birth on maternal morbidity during childbirth hospitalization in California. *Am J Obstet Gynecol* 2015;**213**(5):705.e1-11.
73. Pasupathy D, Wood AM, Pell JP, Fleming M, Smith GC. Time of birth and risk of neonatal death at term: retrospective cohort study. *BMJ* 2010;**341**:c3498.
74. Salihu HM, Ibrahimou B, August EM, Dagne G. Risk of infant mortality with weekend versus weekday births: a population-based study. *J Obstet Gynaecol Res* 2012;**38**(7):973-9.
75. Snowden JM, Darney BG, Cheng YW, McConnell KJ, Caughey AB. Systems factors in obstetric care: the role of daily obstetric volume. *Obstet Gynecol* 2013;**122**(4):851-7.
76. Wu YW, Pham TN, Danielsen B, Towner D, Smith L, Johnston SC. Nighttime delivery and risk of neonatal encephalopathy. *Am J Obstet Gynecol* 2011;**204**(1):37.e1-6.
77. Buckley D, Bulger D. Trends and weekly and seasonal cycles in the rate of errors in the clinical management of hospitalized patients. *Chronobiol Int* 2012;**29**(7):947-54.
78. Givens GH, Smith DD, Tweedie RL. Publication bias in meta-analysis: a Bayesian data-augmentation approach to account for issues exemplified in the passive smoking debate. *Stat Sci* 1997;**12**(4):221-50.
79. Zhou Y, Li W, Herath C, et al. Off-hour admission and mortality risk for 28 specific diseases: a systematic review and meta-analysis of 251 cohorts. *J Am heart Assoc* 2016;**5**(3):e003102.
80. Gupta A, Agarwal R, Ananthakrishnan AN. "Weekend effect" in patients with upper gastrointestinal hemorrhage: a systematic review and meta-analysis. *Am J Gastroenterol* 2017;**14**:14.
81. Sorita A, Ahmed A, Starr SR, et al. Off-hour presentation and outcomes in patients with acute ischemic stroke: a systematic review and meta-analysis. *Eur J Intern Med* 2014;**25**(4):394-400.

82. Sorita A, Ahmed A, Starr SR, et al. Off-hour presentation and outcomes in patients with acute myocardial infarction: systematic review and meta-analysis. *BMJ* 2014;**348**(1):f7393.
